# Neural and behavioral similarity-driven tuning curves for manipulable objects

**DOI:** 10.1162/imag_a_00482

**Published:** 2025-02-18

**Authors:** Daniela Valério, André Peres, Fredrik Bergström, Philipp Seidel, Jorge Almeida

**Affiliations:** Université Paris Saclay, INSERM, CEA, Cognitive Neuroimaging Unit, NeuroSpin Center, Gif-sur-Yvette, France; Collège de France, PSL University, Paris, France; Proaction Laboratory, Faculty of Psychology and Educational Sciences, University of Coimbra, Coimbra, Portugal; CINEICC, Faculty of Psychology and Educational Sciences, University of Coimbra, Coimbra, Portugal; Department of Psychology, University of Gothenburg, Gothenburg, Sweden; Department of Psychiatry and Psychotherapy, Faculty of Medicine, University of Regensburg, Regensburg, Germany

**Keywords:** man-made manipulable objects, tools, release-from-adaptation paradigm, object similarity, fine-grained distinctions

## Abstract

In our daily activities, we encounter numerous objects that we successfully distinguish and recognize within a fraction of a second. This holds for coarse distinctions (e.g., cat vs. hammer) but also for more challenging distinctions that require fine-grain analysis (e.g., cat vs. dog). The efficiency of this recognition depends on how the brain organizes object-related information. While several attempts have focused on unraveling large-scale organization principles, research on fine-grained knowledge organization is rather limited. Here, we explored the fine-grain organization of object knowledge and investigated whether manipulable objects are organized and represented in terms of their similarity. To accomplish this, different groups of individuals participated in a behavioral and functional magnetic resonance imaging (fMRI) release from adaptation experiment. Adaptation was induced by presenting different exemplars of a particular object, and release from adaptation was elicited by the presentation of a deviant object. The relationship between adaptation and deviant objects was manipulated into four levels of similarity, measured by feature overlap between these objects. Our findings revealed that increasing object similarity provoked slower reaction times and weaker fMRI release from adaptation. Specifically, we identified similarity-driven tuning curves for the release from adaptation in the medial fusiform, collateral sulcus, parahippocampal gyri, lingual gyri, lateral occipital complex, and occipito-parietal cortex. These results suggest that the processing and representation of objects in the brain and our ability to perform fine discriminations between objects reflect real-world object similarity in a relatively parametric manner.

## 1 Background

One of the most challenging questions in cognitive neuroscience centers on understanding how we efficiently recognize and distinguish objects. To unravel this ability, we must delve into how information is represented and organized in the brain (e.g.,Almeida, Fracasso, et al., 2023). A widely accepted hypothesis suggests that information might be organized based on similarity in external world dimensions (Edelman, 1998). Examples of this organizational principle abound in sensory-motor cortices—for instance, the visual cortex is organized according to the location of the stimuli in the visual field (i.e., retinotopic maps;Sereno et al., 1995), and the auditory cortex according to the tone of an auditory stimulus (i.e., tonotopic maps;Formisano et al., 2003). In line with this, several studies showed that similarity matters for conceptual representation in the brain—these studies focused on a coarse, between-category, representational level (e.g.,Kriegeskorte et al., 2008). Whether this organization principle extends to finer-grained object distinctions remains unclear. Here, we investigated whether fine-grained similarity shapes the organization of neural and cognitive object representations.

Exploring how object knowledge is represented in the brain has led to the proposal of several hypotheses on the organization of information within the ventral and lateral temporal cortex (Almeida et al., 2013;Caramazza & Shelton, 1998;Chao et al., 1999;Downing & Peelen, 2016;Epstein, 2008;Haxby et al., 2001;Kanwisher et al., 1997;Konkle & Caramazza, 2013;Kriegeskorte et al., 2008;Levy et al., 2001;Mahon & Almeida, 2024;Sha et al., 2015, for a review seeGrill-Spector and Weiner, 2014). For instance, it has been suggested that object knowledge is organized according to coarse domain distinctions, such as animate or inanimate (Caramazza & Shelton, 1998;Kriegeskorte et al., 2008), or more specific categories, such as faces, bodies, animals, manipulable objects, places, among others (Almeida et al., 2013;Chao et al., 1999;Downing & Peelen, 2016;Epstein, 2008;Kanwisher et al., 1997); or that the organization of object knowledge follows specific dimensions such as visual form (Haxby et al., 2001), eccentricity preferences (Arcaro et al., 2019;Levy et al., 2001), real-world object size (Konkle & Caramazza, 2013), animacy (Sha et al., 2015), or is organized according to multidimensional arrangements (Almeida, Fracasso, et al., 2023;Fernandino et al., 2022;Hebart et al., 2020;Huth et al., 2012). Importantly, several of these studies suggested that object similarity in the real world drives the organization of conceptual information in the brain (e.g.,Bracci & Op de Beeck, 2016;Carlson et al., 2014;Fernandino et al., 2022;Huth et al., 2012;King et al., 2019;Kriegeskorte et al., 2008). Moreover, most, if not all, of these studies have tested the role of similarity at a coarse, between-category level.

The number of studies evaluating the fine-grained organization of object knowledge is considerably lower—that is, studies investigating how information is represented among basic level members of a single domain (e.g., within the domain of manipulable objects, how is information represented among the different basic level members such as a knife and a pair of scissors;Almeida, Fracasso, et al., 2023;Almeida, Kristensen, et al., 2023;Bracci et al., 2017;Bruffaerts et al., 2013;Connolly et al., 2012;Gotts et al., 2011;Ruotolo et al., 2020;Xu et al., 2023). This is at odds with the fact that the hardest and perhaps most common everyday decisions we tend to make (e.g., is the object in front of us a knife or a fork) typically rely on finer-grained representations. Moreover, brain-damaged patients with category-specific deficits do not struggle with between-category distinctions (fork vs. dog) but rather with within-category distinctions (fork vs. spoon;Caramazza & Shelton, 1998;Moss & Tyler, 2000). This strongly suggests that unraveling finer-grained levels of representation and, specifically, focusing on whether object similarity is central for within-category object processing is an essential step in understanding object recognition (but see other types of fine-grained organization that take similarity between concepts into account;Bauer & Just, 2017;Rosch et al., 1976).

Here, we focused on the category of manipulable objects to test whether the fine-grained organization conforms to object similarity such that progressive differences in similarity are reflected in behavioral responses and neural representations. To achieve this, we conducted behavioral and functional magnetic resonance imaging (fMRI) experiments using a release from adaptation paradigm, where we parametrically manipulated the similarity between the adaptation and deviant object stimuli. We used this paradigm because it allows the extraction of behavioral and neural similarity-driven tuning curves for manipulable objects. In the behavioral experiment, we measured how quickly and accurately participants detected the deviant object. We expected that the more similar the adaptation and deviant objects were to each other, the harder it would be to differentiate them and the longer the reaction times and the higher the error rates would be. In the fMRI experiment, we measured neural responses to the deviants. We anticipated that some regions would exhibit a differential release (after adaptation) as a function of the similarity between adaptation and deviant objects. Particularly, medial aspects of the ventral temporal cortex, associated with surface and material properties processing (Cant et al., 2009;Cavina-Pratesi et al., 2010a,2010b;Sun et al., 2015), the Lateral Occipital Complex (LOC) involved in processing complex shapes (Grill-Spector et al., 1999), the middle temporal gyrus, responsible for processing functional goals of objects (Lingnau & Downing, 2015;Wurm & Lingnau, 2015), and dorsal occipital cortex and occipito-parietal regions, involved in 3D processing and object manipulation (Cavina-Pratesi et al., 2007;Freud et al., 2016;Jeannerod et al., 1994;Monaco et al., 2011), should show this progressive release from adaptation. As predicted, progressive increases in similarity between adaptation and deviant objects led to significantly slower reaction times and lower recovery of the BOLD signal in the ventral temporal and occipito-parietal regions.

## 2 Experimental Design

For replicability purposes, we ran the behavioral experiment twice (Experiments 1a and 1b), using the same procedures but different object pairs and two independent groups of participants. Experiment 2 involved the fMRI part. For all experiments (1a, 1b, and 2), we selected a set of manipulable objects previously used in our laboratory (Almeida, Fracasso, et al., 2023;Almeida, Kristensen, et al., 2023;Valério et al., 2023). We chose to investigate manipulable objects because (1) these items represent everyday man-made objects that we constantly perceive and interact with; (2) these items encompass different sets of associated information, such as their function, the movements associated with their manipulation, specific structural features (e.g., a handle or rounded shape), and particular contexts in which we use them; (3) this category of objects can be independently impaired (or spared) in patients with brain lesions in the context of spared (or impaired) recognition of items from other domains of knowledge (e.g., animals or faces; for a review seeCapitani et al., 2003); and (4) this category is well suited for using a release from adaptation paradigm because the same object can be rendered in a wide diversity of materials, colors, and shapes (e.g., the various types of glasses). This allows inducing adaptation to an object across different mid-level properties.

To compute between-object similarity, we calculated the cosine similarity formula based on object features and their production frequencies as obtained in a previous study (Valério et al., 2023). In that study, we asked 130 participants to freely generate features for 80 familiar manipulable objects (seeFig. 1A;Valério et al., 2023). Among the concepts used in this study, 36% were visual features (including form, texture, material, and color; e.g., “made of metal”); 24.6% were encyclopedic features (e.g., “found in schools”); 19.8% were functional features (e.g., “used for cutting”); 7.7% were taxonomic features (e.g., “a kitchen object”); 6.3% were action-related features (e.g., “used by putting fingers inside”); 4.9% were tactile features (e.g., “is rough”); and 0.43% were sound-related features (e.g., “is loud”) that contributed to object similarity. SeeSupplementary Table S2for the distribution of these features in the deviant conditions of Experiments 1a and 1b. Note that cosine similarity ranges from 0 to 1, and values closer to 1 indicate high object similarity. Based on these values, we selected pairs of objects according to four similarity levels and binned them together (seeFig. 1A, right). Further details of each experimental design are provided below and can be seen inFigure 1B, C, and D.

**Fig. 1.**
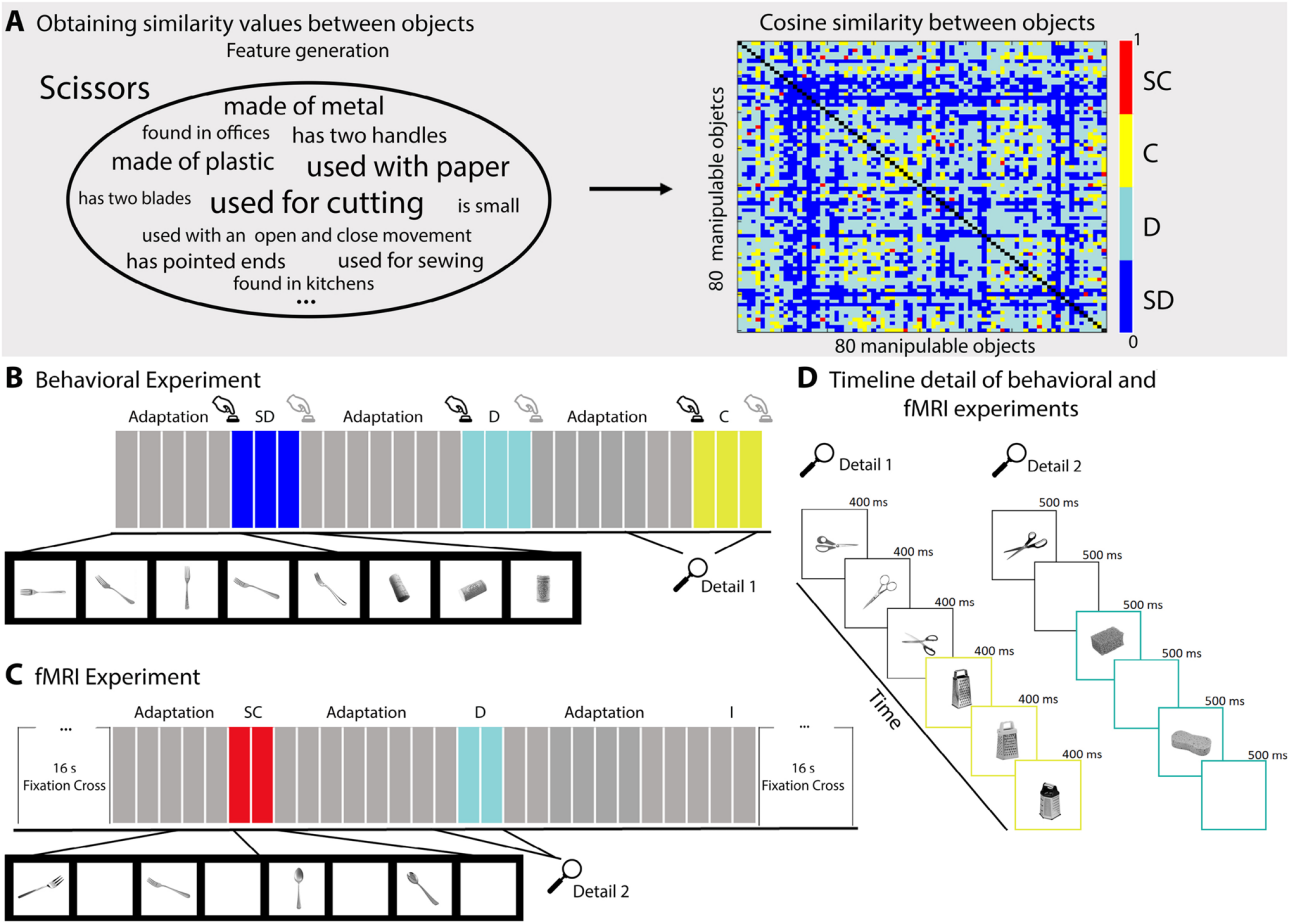
Experimental design. (A) In the gray shadow are the data obtained in a previous study (Valério et al., 2023), illustrating the features generated for pairs of scissors. Using these features, we computed cosine similarity scores among 80 objects, considering their production frequency (the font size in the word cloud represents the production frequency values). In the upper right corner, we present an 80 by 80 matrix of cosine similarity values between an object and all the other 79 objects, color coded according to their similarity. From this matrix, we selected 20 objects—that is, Experiments 1a and 1b—that contained object pairs fitting into 4 levels of similarity: those that were very similar to each other in red– that is, the Super Close bin (SC); those that were similar to each other in yellow—that is, the Close bin; those that were distant from each other in terms of similarity in light blue—that is, the Distant bin (D); and those that were very dissimilar to each other in dark blue—that is, the Super Distant bin (SD). (B) Design of the behavioral experiment. We presented adaptation sequences (i.e., different exemplars of the same object such as a fork) followed by a deviant object. Participants were instructed to press a button whenever there was a change in the object (e.g., from a fork to a cork) but not when the same object was presented under a different perspective or with a different exemplar of the same object (e.g., two different forks). We recorded responses when the object changed from an adaptation sequence to a deviant, but changes from a deviant to a new adaptation sequence were not analyzed (indicated by a gray button press icon). The number of adaptors in an adaptation sequence varied pseudo-randomly between five to nine. Each image was presented for 400 ms, and each deviant type was presented 3 times in a row (i.e., different exemplars of the same object) before another adaptation sequence began. (C) Design of the fMRI experiment. Each run started and finished with 16 s of a screen-centered fixation cross. For each trial, each image was presented for 500 ms followed by 500 ms of a blank screen. The number of adaptors in an adaptation sequence varied pseudo-randomly between five to nine, and each deviant appeared twice. Participants were instructed only to look at the pictures. In both experiments, besides the four experimental manipulations of similarity, we included an Identity condition (I), where the adaptation sequence was immediately followed by a different exemplar of the same object. (D) Two details of the timelines of the behavioral (B) and the fMRI (C) experiments.

## 3 Experiment 1: Behavioral Experiments

### 3.1 Methods

#### 3.1.1 Participants

In Experiment 1a, 22 healthy volunteers (16 females; mean (M) age = 23, standard deviation (SD) age = 6.9, range 18—47; one left handed), and in Experiment 1b, 20 volunteers (13 females, M age = 19.9, SD age = 3.4, range 18–34; all right handed) participated. All participants were naïve about the goal and provided written informed consent. All procedures followed ethical guidelines, and the experiments were approved by the Ethics Committee of the Faculty of Psychology and Educational Sciences of the University of Coimbra in accordance with the Declaration of Helsinki.

#### 3.1.2 Procedures

Experiments 1a and 1b followed the same procedure, each comprising 500 trials, with 50 trials per adaptation object and 10 different adaptation objects (i.e., different exemplars and perspectives of the same object type). For each adaptation object, there were 10 trials for each adaptation length (i.e., 5, 6, 7, 8, or 9 adaptation objects), and within each adaptation length, there were 2 trials for each deviant (i.e., appearing 100 times in total). The five deviant conditions consisted of objects that were very similar to the adaptation object—Super Close (SC) condition; similar to the adaptation object—Close (C) condition; weakly similar to the adaptation object—Distant (D) condition; very weakly similar to the adaptation object—Super Distant (SD) condition, or different exemplars of the same object—Identity condition. Each trial consisted of five to nine exemplars of the same object type (e.g., scissors) that appeared in different perspectives and exemplars (adaptation objects), followed by three exemplars of a deviant object (e.g., graters). For example, take scissors as an adapting stimulus, paired with five deviants: knife represented the SC condition, grater represented the C condition, sponge represented the D condition, match represented the SD condition, and different exemplars of scissors were the Identity condition. Note that the Identity condition was not of interest in this experiment, but was still presented here in order to maintain maximum similarity to the design of Experiment 2. Different pairs of objects were used in Experiment 1 (a and b) for replication purposes with a completely different set of items (and participants)—seeSupplementary Table S1for all pairs used.

All stimuli were grayscale images of objects, with the same object size (400 by 400 pixels, roughly subtending 9.2 degrees of the visual angle). Low-level visual features between adaptation and deviant objects were tested using the Natural Image Statistical Toolbox (Bainbridge & Oliva, 2015). There were no statistical differences between adaptation and deviant objects in terms of luminance and contrast. For high spatial frequency features, there were no significant differences between adaptation and deviant objects, except for the close condition (*p*= 0.04). The results are provided inSupplementary Table S3. Each image was displayed on the screen for 400 ms, with a screen refresh rate of 60 Hz. Participants were instructed to perform a one-back task, where they had to detect when the object changed—that is, participants should press a button, as fast as possible, when the displayed image was of a different object than the immediately preceding one. Responses were collected with a Cedrus button box RB-740, and participants used their dominant hand to respond. We measured accuracy and reaction times, and the experiment lasted for 30 min. Participants were not aware that we only collected responses when the object changed from an adaptor to a deviant (as represented by the black button press icon inFig. 1B), and not vice versa. We used Matlab R2019b and “A Simple Framework” (Schwarzbach, 2011) to present the stimuli.

#### 3.1.3 Statistical analysis

We considered trials with reaction times between 100 and 800 ms as valid. We chose 800 ms as upper limit because, in that case, participants saw two images in a row without pressing the button. Reaction times faster than 100 ms or slower than 800 ms were considered misses. Overall, 2.1% and 3.2% of the total trials in Experiments 1a and 1b were considered misses (either because the reaction times were below or above the cutoffs described above, or because no responses were obtained), respectively. Reaction times and accuracy values were entered in two separate within-subject one-way ANOVAs, with deviant type (the four deviant types of interest) as a factor. Simple effects between deviant type were conducted for each ANOVA if the factor “deviant type” was significant. All comparisons were Bonferroni corrected and conducted in SPSS (version 22, IBM Corp., Armonk, NY, USA, 2013).

### 3.2 Results

For Experiment 1a, reaction times varied significantly as a function of the type of deviant (*F*(3,63) = 52.08;*p*< 0.0001; and partial η² = 0.71). Planned comparisons showed that participants were slower in the SC (mean (M) = 448.5; standard error mean (SEM) = 6.4) condition than in the other conditions (C (*t*(21) = 6.83,*p*< 0.0001), D (*t*(21) = 10.99,*p*< 0.0001), and SD (*t*(21) = 12.18,*p*< 0.0001)); there were no reaction time differences between C (M = 425.6; SEM = 6.6) and D (M = 424.6; SEM = 6.8;*t*(21) = 0.32,*p*= 0.75), but participants were significantly faster in SD (M = 416.7; SEM = 6.7) than in C (*t*(21) = 3.60,*p*= 0.002) and D (*t*(21) = 4.02,*p*= 0.001) conditions. We additionally analyzed the percentage of missing trials (i.e., when participants did not press the button or did so outside of the 100 to 800 ms range). We found that performance varied significantly as a function of the deviant type (*F*(3,63) = 4.91;*p*= 0.004; and partial η² = 0.19). Participants missed more object changes when deviants were part of the SC bin (M = 11.5; SEM = 0.9) than the C (M = 8.4; SEM = 0.9;*t*(21) = 3.05,*p*= 0.006) and SD (M = 8.3; SEM = 0.7;*t*(21) = 2.96,*p*= 0.007) bins. However, we did not find statistical differences between SC and D (*t*(21) = -1.86,*p*= 0.08), C and D (*t*(21) = -1.55,*p*= 0.14), C and SD (*t*(21) = 0.10,*p*= 0.92), or D and SD (*t*(21) = 1.64,*p*= 0.12).

Importantly, in Experiment 1b, we replicated our results with a completely new set of object pairs and an independent group of participants. Performance in reaction time (*F*(3,57) = 29.98;*p*< 0.0001; and partial η² = 0.61) and accuracy (*F*(3,57) = 25.85;*p*< 0.0001; and partial η² = 0.58)) varied significantly by deviant type. Participants were slower in SC (M = 452.3; SEM = 6.6) than in other conditions (C (*t*(19) = 5.44,*p*< 0.0001); D (*t*(19) = 5.22,*p*< 0.0001); and SD (*t*(19) = 8.28,*p*< 0.0001)); there were no differences between C (M = 428.3; SEM = 7.8) and D (M = 433.11; SEM = 7.8;*t*(19) = -1.7,*p*= 0.11) nor between C and SD (M = 422.1; SEM = 7.1;*t*(19) = 2.46,*p*= 0.02) conditions; and participants were significantly faster in SD than in D (*t*(19) = 4.21,*p*< 0.0001) and SC conditions. Moreover, participants were less accurate in SC (M = 19.70; SEM = 1.8) than in C (M = 9.7; SEM = 0.9;*t*(19) = 6.39,*p*< 0.0001), D (M = 10.7; SEM = 1.1;*t*(19) = 5.35,*p*< 0.0001), and SD (M = 8.8; SEM = 0.9;*t*(19) = 5.28,*p*< 0.0001) conditions. However, we did not find statistical differences between C and D (*t*(19) = -1.16,*p*= 0.26), C and SD (*t*(19) = 0.93,*p*= 0.36), and D and SD (*t*(19) = 2.29,*p*= 0.03). All results presented inTable 1andFigure 2were Bonferroni corrected for multiple comparisons. SeeSupplementary Figure S1for the reaction times per type of deviant and participant for Experiments 1 (a and b).

**Table 1:** Reaction times and percentage of missing trials in the behavioral experiments.

		SC		C		D		SD	
		Mean	SEM	Mean	SEM	Mean	SEM	Mean	SEM
Experiment 1a	RTs (ms)	448.5	6.4	425.6	6.6	424.6	6.8	416.7	6.7
	Missings (%)	11.5	0.9	8.4	0.9	9.8	0.8	8.3	0.7
Experiment 1b	RTs (ms)	452.3	6.6	428.3	7.8	433.1	7.8	422.1	7.1
	Missings (%)	19.7	1.8	9.7	0.9	10.7	1.1	8.8	0.9

**Fig. 2.**
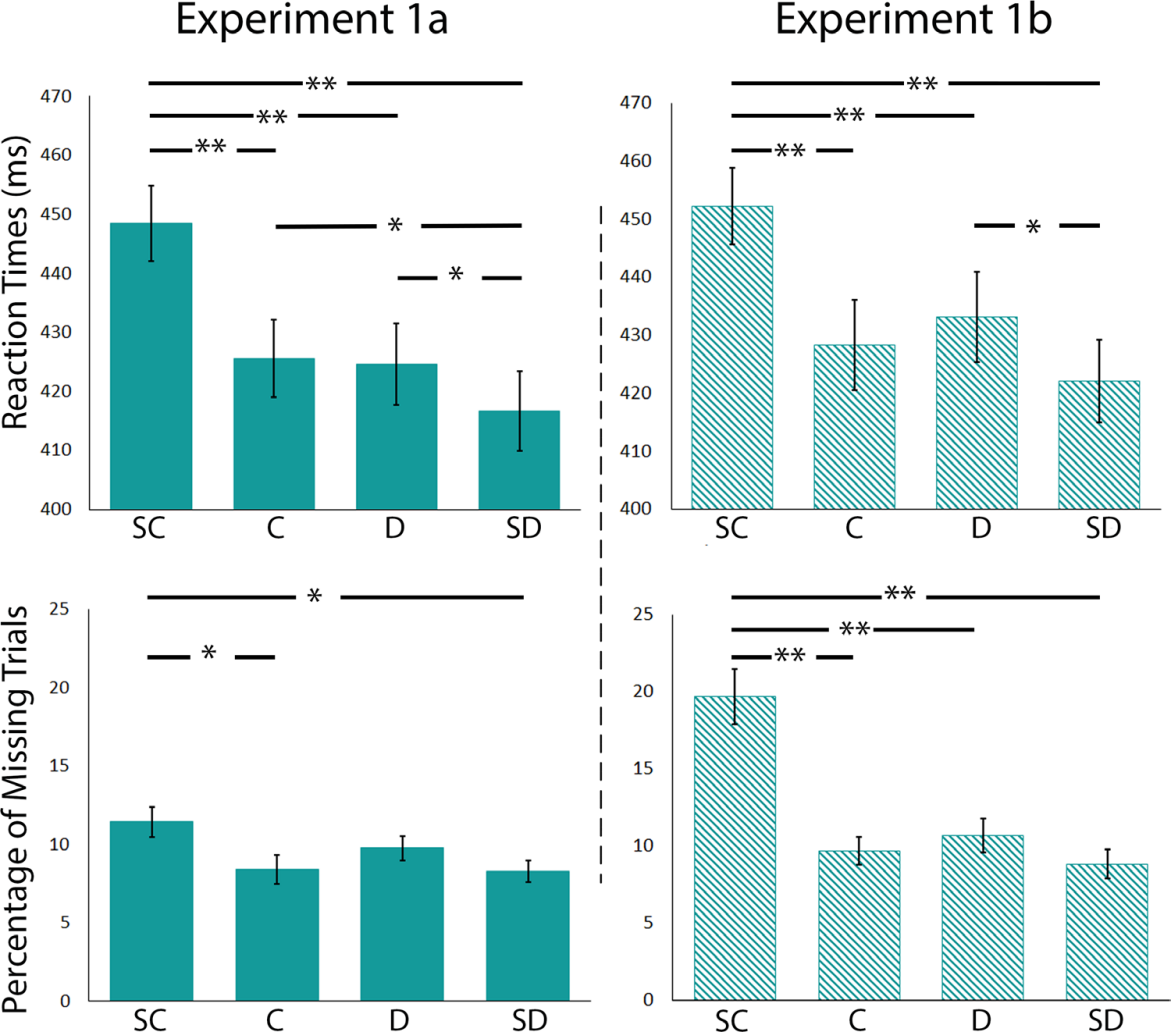
Reaction times and percentage of missing trials for Experiments 1a and 1b. Here we present reaction times and percentage of missing trials as a function of the similarity between the adaptation and deviant objects. Error bars correspond to the standard error of the mean. SC, Super Close; C, Close; D, Distant; SD, Super Distant**p< 0.001, Bonferroni corrected; *p< 0.05, Bonferroni corrected.

### 3.3 Discussion

Overall, as anticipated, Experiments 1 (a and b) revealed that participants are slower and less accurate in detecting object changes when two objects share more features. The results demonstrated a relatively progressive effect of object similarity on object detection reaction times and response accuracy—the further a deviant object is from the adaptation object in terms of object similarity, the easier and faster it is to detect an object change. However, this effect of object similarity presented a plateau in mid-similarity values—that is, similarity effects were more pronounced for the SC deviants, followed by both the C and D deviants, and were weaker for the SD deviants. This will be discussed further in the General Discussion section.

Our behavioral findings suggest that object similarity plays an important role in how our brains organize object concepts, with different levels of similarity affecting this organization. However, the question remains: how does object similarity relate to the neuronal tuning functions of object-preferring regions?

## 4 Experiment 2: fMRI Experiment

To investigate whether object similarity influences the neural organization of object knowledge at a fine-grain representational level, we modified the behavioral experiment for an fMRI setup. Specifically, we again used a release from adaptation paradigm, leveraging the well-known fact that neural signals in regions responsible for a particular type of information adapt after continuous and repeated presentation of that information (Grill-Spector & Malach, 2001;Grill-Spector et al., 2006).

In Experiment 2, we expected that brain areas responsible for processing and storing object knowledge would exhibit two neural phenomena: (1) adaptation (a decrease in the BOLD response) to the repeated presentation of exemplars of the same object type (adaptation objects) and (2) recovery from this adaptation (an increase in BOLD response) when presented with a deviant object. It is important to note that these two analyses are statistically independent, as they are conducted on non-overlapping sets of stimuli and timings of the experiment. Furthermore, what we are interested in is the degree of recovery from adaptation and whether the magnitude of this recovery is a (progressive) function of the similarity between the adaptation and deviant objects—that is, this approach focuses on the voxels that present adaptation and how they recover the BOLD response based on object similarity. What we expect then is that if the neural representation of the adaptation object and the deviant object is extremely similar within a voxel, as expected, for identical or highly related objects with numerous shared features (i.e., highly similar objects), then the recovered response should be relatively weak due to persistent adaptation. Conversely, as we increase dissimilarity between the adaptation and deviant objects in a parametric way, response recovery should progressively increase to non-adapted levels.

### 4.1 Methods

#### 4.1.1 Participants

Twenty-one healthy volunteers (15 females; M age = 25.7, SD age = 5.8, range 18–40) participated in the fMRI experiment. All participants were right handed and had normal or corrected-to-normal vision. Written informed consent was obtained from all participants before starting the study. Participants were monetarily compensated for their participation when they completed the experiment. The study was approved by the Ethics Committee of the Faculty of Psychology and Educational Sciences of the University of Coimbra in accordance with the Declaration of Helsinki. We excluded 5, 4, and 2 runs (out of 10) for 3 participants, respectively, due to excessive head motion, and 2 runs for another participant due to technical problems in the scanner.

#### 4.1.2 Procedures

##### 4.1.2.1 fMRI task

Participants completed two fMRI sessions (separated by at least a week), with five runs in each session. We used an event-related design adapted from Experiment 1a, using the same adaptors and deviants. It is important to note that participants were not instructed to perform the one-back task to avoid response confounds; instead, they were asked to passively view the images. Each run started and ended with 16 s of a centralized fixation cross. Within each run, there were 50 trials, including 10 of each deviant type (SC, C, D, SD, and I) and 10 of each adaptation length (i.e., 5, 6, 7, 8, or 9). Each trial had five to nine adaptation objects, followed by two deviants. All adaptor-deviant pairs were presented in each run. Each stimulus (adaptation or deviant) was presented for 500 ms, followed by 500 ms of a blank screen (seeFig. 1C and D).

Stimulus delivery and response collection were controlled by “A Simple Framework” (Schwarzbach, 2011) based on the Psychophysics Toolbox on Matlab R2019a (The MathWorks Inc., Natick, MA, USA). Stimuli were presented on an Avotec projector with a refresh rate of 60 Hz and viewed by the participants through a mirror attached to the head coil inside the MR scanner bore. For all experiments, we used an eye tracker to (subjectively) monitor the individual’s attention (and wakefulness) during the task. Although participants were completely naïve, we instructed them to pay attention to the stimuli because the experimenter would ask questions at the end. Subsequently, we administered an informal questionnaire of 21 objects and asked them to identify (by crossing them with a pen) the objects they had seen in the scanner. These data were not analyzed and were only collected to ensure that participants were attentive during the task.

##### 4.1.2.2 MR parameters

MRI data were acquired using a 3T MAGNETOM Trio whole-body MR scanner (Siemens Healthineers, Erlangen, Germany) with a 64-channel head coil at the University of Coimbra (Portugal; BIN—National Brain Imaging Network). Structural MRI data were collected using T1-weighted rapid gradient echo (MPRAGE) sequence (repetition time (TR) = 2000 ms, echo time (TE) = 3.5 ms, slice thickness = 1 mm, flip angle = 7 deg, field of view (FoV) = 256 × 256 mm^2^, matrix size = 256 × 256, bandwidth (BW) = 190 Hz/px, GRAPPA acceleration factor 2). Functional MRI (fMRI) data were acquired using a T2*-weighted gradient echo planar imaging (EPI) sequence (TR = 1000 ms, TE = 30 ms, voxel size = 3 × 3 × 3 mm^3^, slice thickness = 3 mm, FoV = 210 × 210 mm^2^, matrix size = 70 × 70, flip angle = 68 deg, BW = 2164 Hz/px, GRAPPA acceleration factor 2), using a multiband factor of 3 (MB = 3). Each image volume consisted of 42 contiguous transverse slices recorded in interleaved ascending slice order oriented parallel to the anterior-posterior commissure plane covering the whole brain. Physiological signals (cardiac and respiratory) were collected.

#### 4.1.3 Data preprocessing

##### 4.1.3.1 Anatomical

Both anatomical and functional data were preprocessed using fMRIPrep 20.2.3. This pipeline includes standard preprocessing steps, organized in an easy-usage workflow that ensures robustness independently of the data idiosyncrasies, and has high consistency of results (Esteban et al., 2019). The T1-weighted (T1w) images were corrected for intensity non-uniformity (INU) with N4BiasFieldCorrection (Tustison et al., 2010), distributed with ANTs 2.3.3 (Avants et al., 2008). The T1w reference was then skull-stripped with a*Nipype*implementation of the ANTS BrainExtraction.sh workflow, using OASIS30ANTs as target template. Brain tissue segmentation of cerebrospinal fluid, white matter, and gray matter was performed on the brain-extracted T1w using fast (FSL 5.0.9;Zhang et al., 2001). A T1w reference map was computed after registration of T1w images (after INU correction) using mri_robust_template (FreeSurfer 6.0.1;Reuter et al., 2010). Brain surfaces were reconstructed using recon-all (FreeSurfer 6.0.1;Dale et al., 1999), and the brain mask estimated previously was refined with a custom variation of the method to reconcile ANTs-derived and FreeSurfer-derived segmentations of the cortical gray matter of Mindboggle (Klein et al., 2017).

##### 4.1.3.2 Functional

A reference volume and its skull-stripped version were generated using custom methodology from*fMRIPrep*. Susceptibility distortion correction was omitted. The BOLD reference was then co-registered to the T1w reference using bbregister (FreeSurfer) which implements boundary-based registration (Greve & Fischl, 2009). Co-registration was configured with six degrees of freedom. Head motion parameters with respect to the BOLD reference (transformation matrices, and six corresponding rotation and translation parameters) were estimated before any spatiotemporal filtering using mcflirt (FSL 5.0.9;Jenkinson et al., 2012). BOLD runs were slice-time corrected using 3dTshift from AFNI (Cox & Hyde, 1997). The BOLD time-series (including slice-timing correction when applied) were resampled onto their original, native space by applying the transforms to correct for head motion. The BOLD time-series was resampled into standard space, generating a*preprocessed BOLD run in MNI152NLin2009cAsym space*. Automatic removal of motion artifacts using independent component analysis (ICA-AROMA;Pruim et al., 2015) was performed on the*preprocessed BOLD on MNI space*time-series after removal of non-steady-state volumes and spatial smoothing with an isotropic, Gaussian kernel of 6 mm FWHM (full-width half-maximum). Corresponding “non-aggressively” denoised runs were produced after such smoothing. The confound time-series derived from head motion estimates were expanded with the inclusion of temporal derivatives and quadratic terms for each (Satterthwaite et al., 2013).

#### 4.1.4 Analysis

##### 4.1.4.1 Adaptation analysis

A random-effects approach within the general linear model in SPM (SPM12—Wellcome Trust Centre for Neuroimaging, London, UK) was used to calculate adaptation effects. There were 14 regressors: 1 per adaptor stimulus (from 1 to 9) and 5 deviant conditions (including the Identity condition). Although we regressed the five deviants, they were not used in this analysis. We included 24 nuisance regressors: 6 motion parameters and 18 physiological regressors of cardiac and respiratory cycles. Nuisance regressors were analyzed using the PhysiO Toolbox in SPM (Kasper et al., 2017), including six cardiac, eight respiratory, and four regressors for the interaction between respiratory and cardiac. We modeled the hemodynamic response for each voxel using a general linear model consisting of finite impulse response (FIR) functions. For each regressor of interest, delta functions representing the response following stimulus presentation at each full-volume echo-planar acquisition in a 1-s window were fitted to the MR signal. This resulted in an estimate of the response to a single stimulus of each stimulus type, with no assumptions about the shape of the hemodynamic response.

Subsequently, we calculated the areas where the activation elicited by the fourth FIR time bin was greater than the seventh (adaptation effect) using an*F*-test for repeated measures—the results were thresholded at*p*< 0.001 (uncorrected). We opted for the seventh FIR time bin instead of the ninth, as only the longest adaptation trials would include the ninth (i.e., allowing us to incorporate 60% of our adaptation trials). Moreover, previous studies have shown that in rapid event-related designs, the hemodynamic response peaks around 4 s after the first image presentation (i.e., in our case, four images in a row), followed by a consistent decrease in the signal before reaching 10 s, after which it stabilizes at a plateau (see the shape of adaptation curves for rapid event-related design inFritsche et al., 2020;Grill-Spector et al., 2006). As a sanity check, we performed the classical contrast of the first FIR time bin greater than the last FIR time bin (*p*< 0.05, uncorrected). The areas showing adaptation were very similar to those in the fourth bigger than the seventh time bins, though with lower activation, as we used only 20% of the trials (SeeSupplementary Fig. S3).

To ensure the validity of our adaptation results, we assessed the areas that presented a greater BOLD activation in response to a different object compared with a different exemplar of the adapted objects. This was achieved by contrasting deviants (SC, C, D, and SD) against the Identity condition. Although we chose not to include this analysis in our main pipeline to avoid potential circularity issues (Kriegeskorte et al., 2009), results are available in theSupplementary Materialand, as expected, the areas obtained with this contrast were similar to the areas that exhibited adaptation as measured above.

##### 4.1.4.2 Release from adaptation analysis

We employed a general linear model in SPM using a hemodynamic response function (HRF) with six regressors of interest: one for all adaptors and one for each deviant. Additionally, we included 24 nuisance regressors: 6 motion parameters and 18 physiological regressors of cardiac and respiratory cycles. Nuisance factors were calculated in the same manner as in the adaptation analysis.

Subsequently, we used the*fslmerge*function (FSL package, version 6.04) to concatenate the betas from the four deviants into a 4D image. It is important to note that we excluded the Identity condition from our analysis as we wanted to see neural tuning curves as a function of similarity between different objects without the confound of having an Identity condition. Then, using Matlab R2021a (The MathWorks Inc., Natick, MA, USA), we calculated a voxel-wise linear regression inside the areas exhibiting adaptation. In other words, we used the areas that showed adaptation effects as a mask, and, within these areas, we searched for voxels exhibiting an increase in BOLD response from SC to SD (i.e., according to our hypothesis). This analysis created an R^2^(i.e., explained variance) map for each participant. Finally, we used the Randomize one-sample*t*-test with 5000 permutations, corrected by threshold-free cluster enhancement (TFCE; FSL package, version 6.04), and controlled for multiple comparisons (*p*FWE-corrected < 0.001). From the areas demonstrating statistically significant R^2^values, we kept the voxels that presented a positive slope. The regions that did not present a positive slope were mainly in the parietal lobe, frontal areas, and the lateral regions of the ventral stream. We decided not to look closely at these that did not exhibit a positive slope because there is no a priori theoretical hypothesis for inspecting these voxels.

##### 4.1.4.3 Cluster analysis

We used cluster analysis to assess whether the similarity-driven tuning curves presented the same shape across all regions that showed a release from adaptation. Initially, we estimated the optimal number of clusters using the Calinski–Harabasz index (Calinski & Harabasz, 1974). Then, we used a k-means clustering algorithm to divide the areas into the number of clusters defined by the index according to the shape of their tuning curves. Since we employed a leave-one-out approach, the division into 2 clusters was repeated 20 times (leaving 1 participant out each time). Then, we extracted the beta activations for each participant using the cluster regions of interest from which that participant had been excluded. For visualizing clusters 1 and 2, we retained the voxels shared by 75% of our sample (i.e., 16 participants; seeFigs. 3Band4D).

**Fig. 3.**
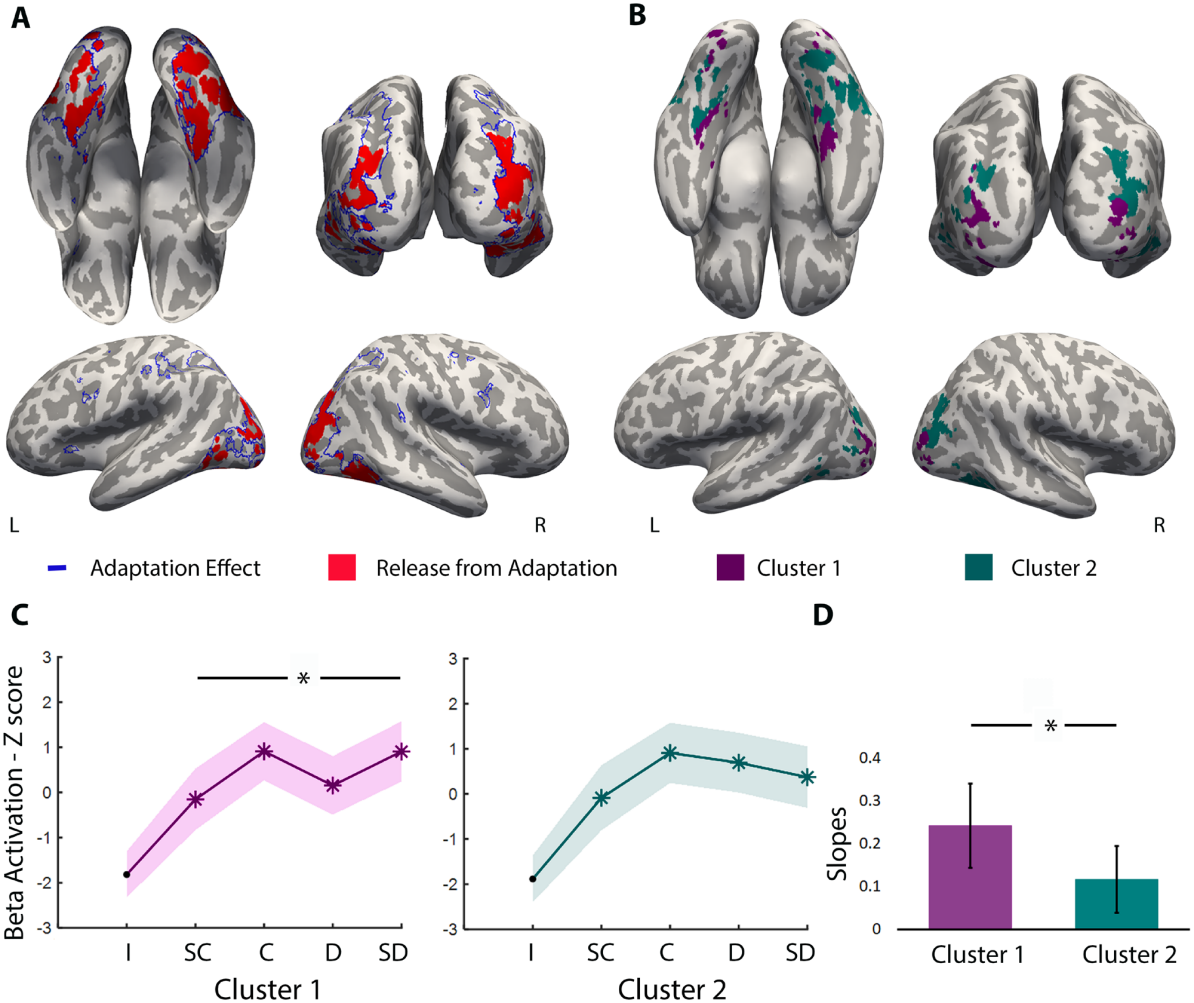
Areas that exhibit adaptation and release from adaptation as a function of similarity between adaptation and deviant objects. (A) The blue outline encompasses areas that exhibit adaptation (4^th^> 7^th^FIR time bins) at a threshold of*p*< 0.001 (uncorrected). Those areas were within ventral occipitotemporal cortex (that goes from LOC to parahippocampal gyrus), parietal lobe (inferior and superior) and occipito-parietal cortex, and frontal lobe (superior, middle, and inferior frontal gyrus, and supplementary motor area). In red, we present areas that show release from adaptation as a function of object similarity (*p*FWE-corrected < 0.001). Bilaterally, these include the collateral sulcus and fusiform gyrus (posterior to anterior), the parahippocampal gyrus, the lingual gyrus, the inferior temporal gyrus, the middle temporal gyrus, LOC, and the most posterior part of the parietal lobe and occipito-parietal cortex. (B) Here, we show the areas corresponding to the 2 clusters that are present in at least 16 participants (75% of our sample). Cluster 1 comprises LOC, occipito-parietal cortex, lingual gyrus, parahippocampal, collateral sulcus, and the most anterior region of medial fusiform; Cluster 2 comprises parts of the fusiform gyrus, middle temporal gyrus, inferior temporal gyrus, and bilateral occipito-parietal cortex, and posterior/caudal IPS. (C) The graphs represent the release effect in BOLD activation (Z-score and SEM) as a function of the four deviants for the areas of cluster 1 (in purple) and cluster 2 (in green; *p< 0.05 Bonferroni corrected). The graphs were Z-scored to enable comparison between adaptation and release. The Identity (I) condition was never used in our analysis, presented here to show visually that beta activation is below the main conditions in the two clusters (in comparison with the deviants,*p*is consistently below 0.05). (D) The graph illustrates the differences in slopes between clusters 1 and 2 using a paired*t*-test (*p= 0.04). SC, Super Close; C, Close; D, Distant; SD, Super Distant.

**Fig. 4.**
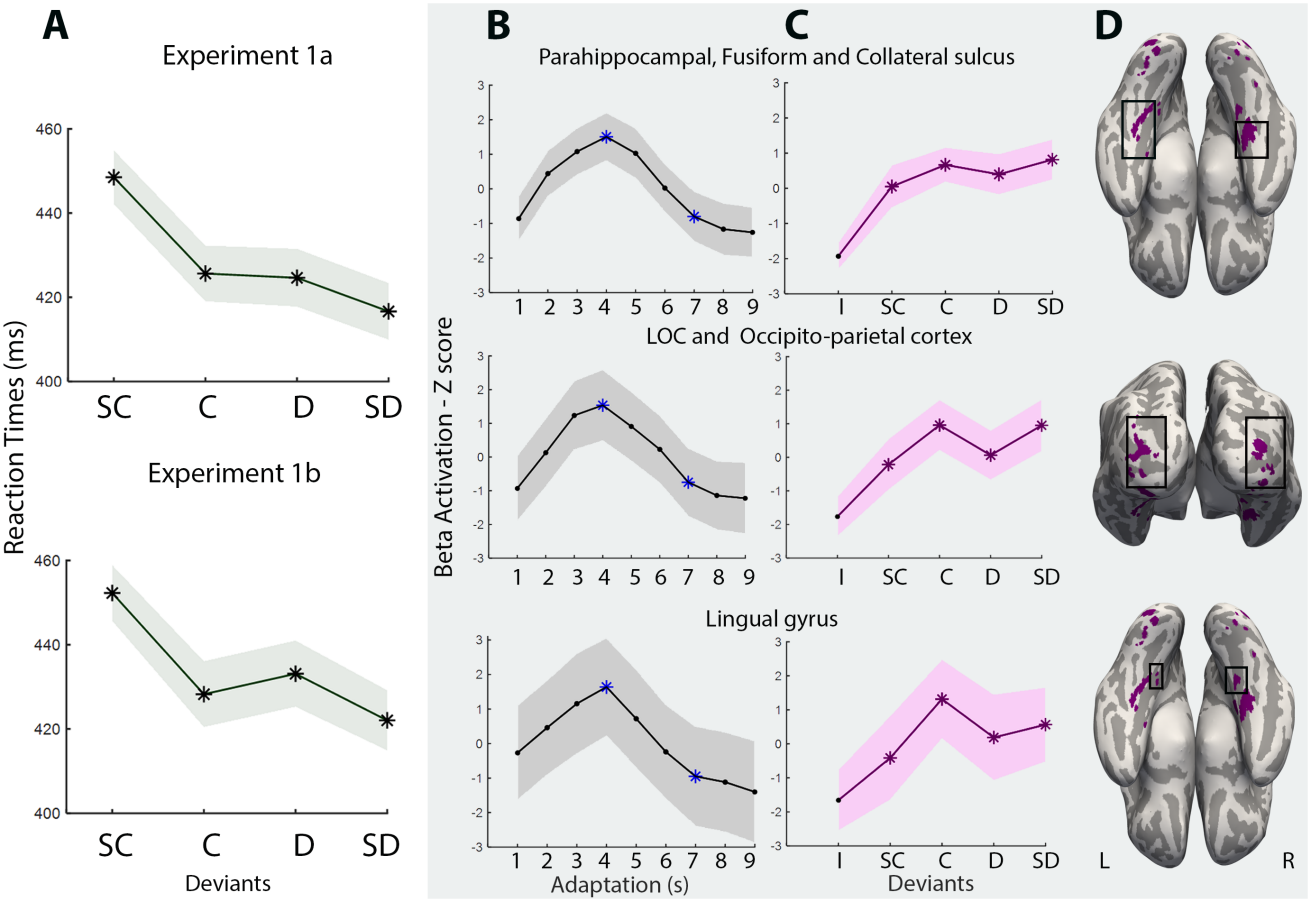
Comparison of the behavioral results and the neural similarity-driven tuning curves for the areas of cluster 1. (A) The results of behavioral reaction times for Experiments 1 (a and b)—the line represents the average, and the shadow represents the SEM. (B) Adaptation curves for each brain area, where we present the beta activation Z-scores. We show the BOLD activation for the nine FIR time bins, and the blue asterisks represent the contrast (4^th^> 7^th^FIR time bins) to measure adaptation. (C) For the deviants, we present the beta-activation Z-scores for the five conditions: Identity (I) was never used in our analysis, but is present to show that beta activation is below the main conditions: Super Close (SC), Close (C), Distant (D), and Super Distant (SD). The graphs were Z-scored to enable comparison between adaptation and release. The asterisks represent the conditions we used to calculate behavioral and neural responses. For columns B and C, the line represents the average BOLD amplitude, and the shadow represents the SEM. (D) Areas of cluster 1.

Subsequently, we Z-transformed the beta activations to compare adaptation and release. Then, for the release analysis, we calculated the slope of the increase from SC to SD for each participant and cluster, and we performed paired*t*-tests to compare the slopes of clusters 1 and 2. Finally, within each cluster, we conducted paired*t*-tests to compare the differences between deviants and corrected them for multiple comparisons using Bonferroni correction. In the cluster graphs, we present the Identity condition beta activation to illustrate that it is lower than the beta activations for the other four deviants.

### 4.2 Results

We first determined which regions showed a decrease in BOLD response to the adaptation objects. We computed a univariate whole-brain contrast to identify voxels where the fourth adaptation stimulus elicited stronger responses than the seventh—that is, searching for those areas that showed a decrease in BOLD response due to the repetition of (different exemplars and/or perspectives of) an object type (e.g., a fork; seeSupplementary Fig. S3for the adaptation curves).

As depicted inFigure 3A, we observe neural adaptation in regions within the ventral occipitotemporal cortex bilaterally, extending from the LOC to the parahippocampal gyrus, and dorsal occipital regions extending to the superior and inferior parietal cortex and the intraparietal sulcus (IPS). There are also more sparse adaptation effects within the superior, middle, and inferior frontal gyri and supplementary motor area (seeFig. 3A; blue outline—adaptation effect). To confirm these results, we also contrasted the deviants (SC, C, D, and SD) with the Identity condition (seeSupplementary Fig. S2), and the areas that adapted were very similar. In subsequent analyses, we focused on the adaptation regions obtained with the former method (i.e., the adaptation period; 4^th^> 7^th^FIR time bins).

We then tested which of these regions showed graded release from this adaptation as a function of object similarity. To do so, we first computed a voxel-wise linear regression (not including the Identity condition) in search for voxels that showed an increase in release from adaptation from the SC to the SD condition. As can be seen inFigure 3A(in red), release from adaptation is present in medial aspects of the ventral temporal cortex along the collateral sulcus, posterior to anterior, from the lingual gyrus to the parahippocampal gyrus. We also observed release from adaptation within the lateral temporal cortex, in the posterior middle temporal gyrus, and finally, in the dorsal occipital and posterior parietal cortical regions. All the release from adaptation effects was bilateral (seeFig. 3A, red areas).

Subsequently, we evaluated the characteristics of the neuronal tuning curves in regions showing release from adaptation. We split the data into two clusters according to the Calinski–Harabasz optimal number and applied k-means clustering with two centroids, revealing two distinct similarity-driven tuning curves. Cluster 1 included voxels bilaterally within the medial fusiform, collateral sulcus, parahippocampal gyrus, LOC, and occipito-parietal cortex (seeFig. 3B and Cin purple). Cluster 2 showed average tuning curves that are more modest in their release from adaptation, including voxels in more lateral areas of the fusiform gyrus, occipito-temporal, occipito-parietal cortex, and caudal IPS (seeFig. 3B and Cin green). Beta values were extracted for each participant and deviant type, and we compared the slopes between clusters and the beta values across deviants for each cluster. We identified that one participant exhibited beta values 3 standard deviations greater than the group average. Thus, this participant was identified as an outlier, and consequently excluded from these comparisons. However, the results, including this outlier participant, are available in the Supplementary Material (seeSupplementary Fig. S4; for whole brain similarity-based graded release maps, seeSupplementary Fig. S5). In an exploratory analysis, we compared the slope of cluster 1 (M = 0.24; SEM = 0.1) and cluster 2 (M = 0.12; SEM = 0.08) using a paired*t*-test (one-tailed) and found that cluster 1 is statistically steeper than cluster 2 (*t*(19) = 1.82,*p*= 0.04; seeFig. 3D). Using a non-parametric test—the Wilcoxon Signed-Rank test, we confirmed that cluster 1 is statistically steeper than cluster 2 (*Z*= -2.05,*p*= 0.04). That is, areas in cluster 1 show a progressive release from adaptation as one traverses the different levels of similarity between the adaptation and deviant objects from very similar to very dissimilar. Moreover, Z-score beta activation for cluster 1 indicated statistically lower values for SC (M = -0.15, SEM = 0.68) than SD (M = 0.91; SEM = 0.67;*t*(19) = -3.32,*p*= 0.004; two-tailed; seeFig. 3C). Other comparisons (two-tailed) were not statistically significant after Bonferroni correction: D and SD (M = 0.15; SEM = 0.65;*t*(19) = -1.95,*p*= 0.07), SC and C (M = 0.91; SEM = 0.64;*t*(19) = -2.70,*p*= 0.01), SC and D (*t*(19) = -0.75,*p*= 0.46), C and D (*t*(19) = 2.08,*p*= 0.05), C and SD (*t*(19) = 0.01,*p*= 0.99). In cluster 2, there were no statistically significant results after correction for multiple comparisons.

Importantly, the release observed in cluster 1 seems to align more closely with the behavioral data obtained, as it exhibits a steeper slope and a significant difference between SC and SD. Therefore, we focused more on this cluster. InFigure 4, we present the adaptation and release graphs for the areas within cluster 1. Additionally, we include the behavioral results from Experiments 1a and 1b to visually demonstrate the congruency between behavioral and neural data. Interestingly, our results suggest a possible inverse relationship between the behavioral and neural findings. When two objects were more similar (i.e., SC condition), participants tended to be slower and less accurate in detecting the object, and this was reflected in a weaker release effect at the neural level. In contrast, when two objects were more distinct (i.e., SD condition), participants tended to be faster and more accurate behaviorally, and there was a stronger release effect at the neural level.

### 4.3 Discussion

In Experiment 2, as expected, we observed a gradual reduction in BOLD activation due to the repetitive presentation of an adapting stimulus (different exemplars and/or perspectives of the same object). The bilateral areas demonstrating adaptation effects included the occipito-temporal cortex, parietal lobe, occipito-parietal cortex, frontal areas (superior, middle, and inferior frontal gyri), and supplementary motor area). These areas are known to be preferentially activated by manipulable objects (e.g.,Almeida et al., 2013;Bergström et al., 2021;Chao et al., 1999;Mahon et al., 2007). Importantly, we found a release in the BOLD response as a function of object similarity in some areas that presented adaptation. These areas encompassed the fusiform gyrus, collateral sulcus, parahippocampal gyrus, lingual gyrus, inferior temporal gyrus, middle temporal gyrus, LOC, the most posterior part of the parietal lobe, and occipito-parietal cortex (seeFig. 3A). Voxels within these areas appear to be tuned to object similarity.

Importantly, these different areas are organized into two distinct clusters based on the tuning curves for the similarity between the adaptation and deviant stimuli. Particularly, the medial fusiform gyrus, collateral sulcus, parahippocampal gyrus, the ventral part of LOC, occipito-parietal cortex, and lingual gyrus formed one cluster (cluster 1; seeFig. 4B, C, and D). This cluster revealed a clear distinction between SC and SD and seems to align more closely with our behavioral data in Experiments 1 (a and b). In contrast, ventral temporal areas situated more posterior and lateral than those in cluster 1, as well as dorsal occipital regions that are more superior and anterior than those in cluster 1, belonged to another cluster as a function of their tuning function (cluster 2). These areas displayed a more modest tuning response to changes in object similarity—visually, there appears to be a distinction between the SC condition and all other deviants in this cluster (seeFig. 3B and C). These differences may relate to the processing preferences of the clustered areas and, potentially, the processing level at which these areas operate.

## 5 General Discussion

Our ability to recognize and discriminate one object from other objects in the environment is crucial for completing basic daily tasks. To understand this ability, we may need to unravel how object knowledge is organized in the brain—and, specifically, we may need to focus on finer-grain representations and test whether, at this level of representation, similarity is a major organizing principle of information in the brain (Grill-Spector & Weiner, 2014;Op De Beeck et al., 2008). Here, we demonstrate that object similarity might impact fine-grained object representations, with similar objects being represented more closely than more dissimilar ones. This may reflect a pattern similar to how sensory-motor regions encode low-level perceptual similarities. This observation seems to be particularly true for the medial fusiform gyrus and collateral sulcus, the parahippocampal gyrus, the lingual gyrus, and LOC—that is, major ventral stream regions important for object recognition and processing (Grill-Spector & Weiner, 2014)—as well as occipito-parietal cortex.

Behaviorally, we demonstrated that participants faced greater difficulty in detecting an object change when the adaptation object and the deviant object were more similar. This implies that detecting an object becomes more challenging in the presence of similar objects, suggesting higher confusability between objects with greater object similarity. Analogous results have been consistently observed in numerical processing. Specifically, performance in a number decision task—for example, deciding whether a target numerosity set was different than the preceding one—varied as a function of the distance between the two sets. Parenthetically, it has been shown that number similarity, described by Weber’s law, governs neural tuning curves in the intraparietal sulcus (Harvey et al., 2013;Kersey & Cantlon, 2017;Piazza et al., 2004).

Notably, our results show that neural responses in regions of cluster 1 (i.e., parahippocampal and medial fusiform gyri, collateral sulcus, lingual gyri, LOC, and occipito-parietal cortex) may be sensitive to the full spectrum of object similarity. Moreover, the shape of their release tuning curve aligns with that obtained in our behavioral experiments, suggesting some involvement in our ability to detect and recognize a different object—that is, the increasing similarity between two deviant and adaptation objects led to slower reaction times and weaker recovery of the BOLD response. Areas of this cluster have been shown to be involved in aspects of high-level manipulable object processing. Specifically, they play a crucial role in processing object-specific visual information, such as information on shape, material, and surface properties (Almeida, Fracasso, et al., 2023;Cant et al., 2009;Cant & Goodale, 2007;Freud, Ganel, et al., 2017;Gallivan et al., 2014;Grill-Spector & Malach, 2001;Hayworth & Biederman, 2006;Pyles & Grossman, 2009). Additionally, the medial fusiform gyrus has been associated with motor-relevant object information and functional grasping (Almeida, Fracasso, et al., 2023;Chen et al., 2016;Knights et al., 2022;Lee et al., 2019;Mahon & Almeida, 2024;Mahon et al., 2007;Valyear & Culham, 2010), whereas the parahippocampal and fusiform gyri have an important role in processing semantic knowledge and object function (Almeida, Fracasso, et al., 2023;Aminoff et al., 2013;Chao et al., 1999;Kleineberg et al., 2018;Mandera et al., 2017;Wheatley et al., 2005). In fact, the medial fusiform gyrus might work as a central area for processing information about manipulable objects and shows privileged functional connectivity with the parietal lobe, premotor cortex, and lateral occipitotemporal cortex (Almeida et al., 2013;Amaral et al., 2021;Chen et al., 2017;Garcea et al., 2016;Kristensen et al., 2016;Mahon & Caramazza, 2011;Mahon et al., 2007,^2013^;Stevens et al., 2015;Walbrin & Almeida, 2021), for a functional description of this connectivity, seeMahon & Almeida (2024). Finally, occipito-parietal regions within this cluster are involved in object-specific 3D processing (Almeida, Fracasso, et al., 2023;Almeida et al., 2014;Freud et al., 2016;Georgieva et al., 2008;Kilintari et al., 2011) and object grasping (Almeida, Fracasso, et al., 2023;Davare et al., 2011;Jeannerod et al., 1994;Monaco et al., 2011).

A second cluster of areas shows a more modest release from adaptation and seems to have only a very crude representation of similarity. This cluster includes areas more posterior and superior to those in cluster 1, such as the posterior occipital–temporal and superior occipito-parietal cortex, which are also involved in processing shape, texture, and motor functions (Almeida, Fracasso, et al., 2023;Freud, Culham, et al., 2017;Peuskens et al., 2004;Wurm & Lingnau, 2015). However, these regions potentially encode less complex and lower-level features, which might not allow for fine-grain representations necessary for fully differentiating objects or for encoding a comprehensive object space. Interestingly, the ventral temporal regions associated with clusters 1 and 2 seem to follow an organization from anterior and medial to posterior and lateral: regions in cluster 1 are more medial and anterior, whereas regions in cluster 2 are more posterior and lateral. Perhaps this posterior-to-anterior difference reflects the putative hierarchical processing of objects and how this processing relates to increasingly fine-grained processing of object features and object knowledge. On the one hand, lower levels of analysis, whereby very crude processing of object knowledge leads to rather rudimental similarity effects (i.e., making it harder to see graded similarity differences), are present in more posterior and lateral regions. On the other hand, as the level of complexity of analysis increases and we move more anteriorly and medially, the processing of object features becomes more fine-grained, and concomitant feature-based object similarity becomes more apparent.

The role of similarity in fine-grained representation likely follows the same principles as those governing coarse-level representations. For instance, the well-known distinction between animate and inanimate objects may arise because animate members are more similar than those of the inanimate category (Grill-Spector & Weiner, 2014;Kriegeskorte et al., 2008). Within brain regions that represent and process manipulable objects, information about specific objects (e.g., scissors) might be represented along one or more dimensions following their similarity with the real world. Interestingly, at a more fine-grained level,Bauer and Just (2017)found that basic concepts (e.g., scissors) appear to be more neurally similar to their typical subordinates (e.g., paper scissors) than to their atypical subordinates (e.g., pruning shears).

A key aspect of this paper involves the use of a release from adaptation technique. Many current studies on similarity have used multivariate approaches across stimuli to explore the role of similarity in object processing (e.g.,Carlson et al., 2014;Contini et al., 2020;Fernandino et al., 2022;Kriegeskorte et al., 2008). However, we firmly believe that this technique is the most suitable design to measure similarity-based tuning curves and show the graded nature of similarity as a principle in organizing finer-grained representations—as potentially seen in other types of information processing (Fabbri et al., 2010;Piazza et al., 2004;Planton et al., 2021). The nature of this technique allows us to see individual effects of progressive increase in object similarity in the processing of objects. Moreover, by using release from adaptation, we can conduct behavioral and neural experiments with a similar structure, facilitating the comparison of reaction times and BOLD recovery. Additionally, adaptation is likely a mechanism that aids in conserving energy and enhances the sensitivity of the neural population to new stimuli (Grill-Spector et al., 2006), making release from adaptation probably more sensitive to fine-grained distinctions. Studies using adaptation have significantly contributed to our understanding of how fine-grained information is represented in the human brain. One of the most prominent examples is the numerosity studies (Cantlon et al., 2006;Piazza et al., 2004,^2007^), which paved the way for later ground-breaking discoveries, such as identifying the numerosity maps in the intraparietal sulcus (Harvey et al., 2013). Note that although adaptation and release from adaptation techniques are still widely used nowadays, some authors have raised valid concerns about the challenges in interpreting these effects (Larsson et al., 2016). For example, these challenges include the still-unknown origin of the neural adaptation phenomenon, the difficulty in determining whether adaptation occurs in the imaged brain area or reflects effects inherited from upstream areas and the possibility that the results may reflect in the vascular activity (Larsson et al., 2016).

Importantly, in our behavioral and neural tuning curves, particularly in areas within cluster 1, there is a clear distinction between SC and SD within the category of manipulable objects. However, the results are less conclusive at intermediate levels (i.e., C and D). With only four levels of similarity (i.e., SC, C, D, and SD) and without a full spectrum, it is impossible to determine the precise shape of the tuning curve or confidently say that it exhibits a graded effect. Our data seem to suggest that the complete tuning curve may not be entirely linear, possibly because the similarity values in these conditions are not far enough apart to result in appreciable behavioral and neural differences (seeSupplementary Table S1). One potential solution for addressing these intermediate levels is to conduct the same experiments with more deviants to obtain enough data points for curve fitting. Nevertheless, the results of both experiments present evidence of release from adaptation consistent with strong cognitive and neural tuning functions for fine-grained object similarity.

Finally, we purportedly employed a relatively broad measure of similarity that combined all object features irrespective of their content. As such, we cannot say, with certainty, what types of content are driving similarity effects in behavioral responses or the similarity releases in the different brain areas. Although we acknowledge the involvement of these areas in visual processing (Cant et al., 2009;Cant & Goodale, 2007;Freud, Ganel, et al., 2017;Gallivan et al., 2014;Grill-Spector & Malach, 2001;Hayworth & Biederman, 2006;Pyles & Grossman, 2009), we consider it unlikely that we are only assessing this aspect, especially, because several studies have shown that these same areas might be central for processing various others types of knowledge (Almeida, Fracasso, et al., 2023;Aminoff et al., 2013;Chao et al., 1999;Chen et al., 2016;Knights et al., 2022;Lee et al., 2019;Mahon et al., 2007;Mandera et al., 2017;Valyear & Culham, 2010;Wheatley et al., 2005). In fact, while visual features greatly influence object similarity, contributing 36%, other aspects also play important roles, such as encyclopedic and functional features, accounting for 24.6% and 19.8% of the features, respectively. Additionally, our adaptation paradigm presented different exemplars and perspectives of the same object, leading us to believe that participants were adapting to the object itself and not specifically to low- to mid-level features such as orientation. Thus, in the same vein, the recovery of the BOLD response should depend not only on these low- to mid-level features but also on other significant object properties (visual or not), such as texture, material, action knowledge, or other conceptual information. However, a limitation of our study is the lack of a scrambled version of the deviants, which would have helped to rule out the effect of low-level features, but at the same time would have doubled the number of trials.

Overall, our results might suggest that information about manipulable objects is represented in such a way as to maintain real-object similarity in a relatively graded way, both cognitively and neurally. This supports the hypothesis that object similarity reflects real-world proximity and that finer-grained representations within a category might retain a rather detailed and graded map of similarity. This representation likely plays a central role in how the brain processes and recognizes everyday objects.

## Supplementary Material

Supplementary Material

## Data Availability

fMRI data (raw and preprocessed) are available on OpenNeuro (https://openneuro.org/datasets/ds005891/versions/1.0.0). Behavioral and fMRI main outputs and the codes are available on OSF (https://osf.io/3vgq2/).

## References

[b1] Almeida , J. , Fintzi , A. R. , & Mahon , B. Z . ( 2013 ). Tool manipulation knowledge is retrieved by way of the ventral visual object processing pathway . Cortex , 49 ( 9 ), 2334 – 2344 . 10.1016/j.cortex.2013.05.004 23810714 PMC3795789

[b2] Almeida , J. , Fracasso , A. , Kristensen , S. , Valério , D. , Bergström , F. , Chakravarthi , R. , Tal , Z. , & Walbrin , J . ( 2023 ). Neural and behavioral signatures of the multidimensionality of manipulable object processing . Communications Biology , 6 ( 1 ), 1 – 16 . 10.1038/s42003-023-05323-x 37709924 PMC10502059

[b3] Almeida , J. , Kristensen , S. , Tal , Z. , & Fracasso , A . ( 2023 ). Contentopic mapping in ventral and dorsal association cortex: The topographical organization of manipulable object information . BioRxiv . 10.1101/2023.11.29.568856 PMC12586957 41067668

[b4] Almeida , J. , Mahon , B. Z. , Zapater-Raberov , V. , Dziuba , A. , Cabaço , T. , Marques , J. F. , & Caramazza , A . ( 2014 ). Grasping with the eyes: The role of elongation in visual recognition of manipulable objects . Cognitive, Affective and Behavioral Neuroscience , 14 ( 1 ), 319 – 335 . 10.3758/s13415-013-0208-0 23996788

[b5] Amaral , L. , Bergström , F. , & Almeida , J . ( 2021 ). Overlapping but distinct: Distal connectivity dissociates hand and tool processing networks . Cortex , 140 , 1 – 13 . 10.1016/j.cortex.2021.03.011 33901719

[b6] Aminoff , E. M. , Kveraga , K. , & Bar , M . ( 2013 ). The role of the parahippocampal cortex in cognition . Trends in Cognitive Sciences , 17 ( 8 ), 379 – 390 . 10.1016/j.tics.2013.06.009 23850264 PMC3786097

[b7] Arcaro , M. J. , Schade , P. F. , & Livingstone , M. S . ( 2019 ). Universal mechanisms and the development of the face network: What you see is what you get . Annual Review of Vision Science , 5 ( 1 ), 341 – 372 . 10.1146/annurev-vision-091718-014917 PMC7568401 31226011

[b8] Avants , B. B. , Epstein , C. L. , Grossman , M. , & Gee , J. C . ( 2008 ). Symmetric diffeomorphic image registration with cross-correlation: Evaluating automated labeling of elderly and neurodegenerative brain . Medical Image Analysis , 12 ( 1 ), 26 – 41 . 10.1016/j.media.2007.06.004 17659998 PMC2276735

[b9] Bainbridge , W. A. , & Oliva , A . ( 2015 ). A toolbox and sample object perception data for equalization of natural images . Data in Brief , 5 , 846 – 851 . 10.1016/j.dib.2015.10.030 26693521 PMC4660227

[b10] Bauer , A. J. , & Just , M. A . ( 2017 ). A brain-based account of “basic-level” concepts . NeuroImage , 161 , 196 – 205 . 10.1016/j.neuroimage.2017.08.049 28826947 PMC5696099

[b11] Bergström , F. , Wurm , M. , Valério , D. , Lingnau , A. , & Almeida , J . ( 2021 ). Decoding stimuli (tool-hand) and viewpoint invariant grasp-type information . Cortex , 139 , 152 – 165 . 10.1016/j.cortex.2021.03.004 33873036

[b12] Bracci , S. , Daniels , N. , & De Beeck , H. O . ( 2017 ). Task context overrules object- and category-related representational content in the human parietal cortex . Cerebral Cortex , 27 ( 1 ), 310 – 321 . 10.1093/cercor/bhw419 28108492 PMC5939221

[b13] Bracci , S. , & Op de Beeck , H . ( 2016 ). Dissociations and associations between shape and category representations in the two visual pathways . Journal of Neuroscience , 36 ( 2 ), 432 – 444 . 10.1523/JNEUROSCI.2314-15.2016 26758835 PMC6602035

[b14] Bruffaerts , R. , Dupont , P. , Peeters , R. , De Deyne , S. , Storms , G. , & Vandenberghe , R . ( 2013 ). Similarity of fMRI activity patterns in left perirhinal cortex reflects semantic similarity between words . Journal of Neuroscience , 33 ( 47 ), 18597 – 18607 . 10.1523/JNEUROSCI.1548-13.2013 24259581 PMC6618797

[b15] Calinski , T. , & Harabasz , J . ( 1974 ). A dendrite method for cluster analysis . Communications in Statistics , 3 ( 1 ), 1 – 27 . 10.1080/03610927408827101

[b16] Cant , J. S. , Arnott , S. R. , & Goodale , M. A . ( 2009 ). fMR-adaptation reveals separate processing regions for the perception of form and texture in the human ventral stream . Experimental Brain Research , 192 ( 3 ), 391 – 405 . 10.1007/s00221-008-1573-8 18815774

[b17] Cant , J. S. , & Goodale , M. A . ( 2007 ). Attention to form or surface properties modulates different regions of human occipitotemporal cortex . Cerebral Cortex , 17 ( 3 ), 713 – 731 . 10.1093/cercor/bhk022 16648452

[b18] Cantlon , J. F. , Brannon , E. M. , Carter , E. J. , & Pelphrey , K. A . ( 2006 ). Functional imaging of numerical processing in adults and 4-y-old children . PLoS Biology , 4 ( 5 ), 844 – 854 . 10.1371/journal.pbio.0040125 PMC1431577 16594732

[b19] Capitani , E. , Laiacona , M. , Mahon , B. , & Caramazza , A . ( 2003 ). What are the facts of semantic category-specific deficits? A critical review of the clinical evidence . Cognitive Neuropsychology , 20 ( 3–6 ), 213 – 261 . 10.1080/02643290244000266 20957571

[b20] Caramazza , A. , & Shelton , J. R . ( 1998 ). Domain-specific knowledge systems in the brain: The animate-inanimate distinction . Journal of Cognitive Neuroscience , 10 ( 1 ), 1 – 34 . 10.1162/089892998563752 9526080

[b21] Carlson , T. A. , Simmons , R. A. , Kriegeskorte , N. , & Slevc , L. R . ( 2014 ). The emergence of semantic meaning in the ventral temporal pathway . Journal of Cognitive Neuroscience , 26 ( 1 ), 120 – 131 . 10.1162/jocn_a_00458 23915056

[b22] Cavina-Pratesi , C. , Goodale , M. A. , & Culham , J. C . ( 2007 ). FMRI reveals a dissociation between grasping and perceiving the size of real 3D objects . PLoS One , 2 ( 5 ), 1 – 14 . 10.1371/journal.pone.0000424 PMC1855433 17487272

[b23] Cavina-Pratesi , C. , Kentridge , R. W. , Heywood , C. A. , & Milner , A. D . ( 2010a ). Separate channels for processing form, texture, and color: Evidence from fMRI adaptation and visual object agnosia . Cerebral Cortex , 20 ( 10 ), 2319 – 2332 . 10.1093/cercor/bhp298 20100900

[b24] Cavina-Pratesi , C. , Kentridge , R. W. , Heywood , C. A. , & Milner , A. D . ( 2010b ). Separate processing of texture and form in the ventral stream: Evidence from fMRI and visual agnosia . Cerebral Cortex , 20 ( 2 ), 433 – 446 . 10.1093/cercor/bhp111 19478035

[b25] Chao , L. L. , Haxby , J. V , & Martin , A . ( 1999 ). Attribute-based neural substrates in temporal cortex for perceiving and knowing about objects . Nature Neuroscience , 2 ( 10 ), 913 – 919 . 10.1038/13217 10491613

[b26] Chen , Q. , Garcea , F. E. , Almeida , J. , & Mahon , B. Z . ( 2017 ). Connectivity-based constraints on category-specificity in the ventral object processing pathway . Neuropsychologia , 105 , 184 – 196 . 10.1016/j.neuropsychologia.2016.11.014 27876509 PMC5438294

[b27] Chen , Q. , Garcea , F. E. , & Mahon , B. Z . ( 2016 ). The representation of object-directed action and function knowledge in the human brain . Cerebral Cortex , 26 ( 4 ), 1609 – 1618 . 10.1093/cercor/bhu328 25595179 PMC4785951

[b28] Connolly , A. C. , Guntupalli , J. S. , Gors , J. , Hanke , M. , Halchenko , Y. O. , Wu , Y. C. , Abdi , H. , & Haxby , J. V . ( 2012 ). The representation of biological classes in the human brain . Journal of Neuroscience , 32 ( 8 ), 2608 – 2618 . 10.1523/JNEUROSCI.5547-11.2012 22357845 PMC3532035

[b29] Contini , E. W. , Goddard , E. , Grootswagers , T. , Williams , M. , & Carlson , T . ( 2020 ). A humanness dimension to visual object coding in the brain . NeuroImage , 221 ( 117139 ), 1 – 11 . 10.1016/j.neuroimage.2020.117139 32663643

[b30] Cox , R. W. , & Hyde , J. S . ( 1997 ). Software tools for analysis and visualization of fMRI data . NMR in Biomedicine: An International Journal Devoted to the Development and Application of Magnetic Resonance In Vivo , 10 ( 4–5 ), 171 – 178. 10.1002/(SICI)1099-1492(199706/08)10:4/5<171::AID-NBM453>3.0.CO;2-L 9430344

[b31] Dale , A. M. , Fischl , B. , & Sereno , M. I . ( 1999 ). Cortical surface-based analysis . NeuroImage , 9 ( 2 ), 179 – 194 . 10.1006/nimg.1998.0395 9931268

[b32] Davare , M. , Kraskov , A. , Rothwell , J. C. , & Lemon , R. N . ( 2011 ). Interactions between areas of the cortical grasping network . Current Opinion in Neurobiology , 21 ( 4 ), 565 – 570 . 10.1016/j.conb.2011.05.021 21696944 PMC3437559

[b33] Downing , P. E. , & Peelen , M. V . ( 2016 ). Body selectivity in occipitotemporal cortex: Causal evidence . Neuropsychologia , 83 , 138 – 148 . 10.1016/j.neuropsychologia.2015.05.033 26044771

[b34] Edelman , S . ( 1998 ). Representation is representation of similarities . Behavioral and Brain Sciences , 21 ( 4 ), 449 – 498 . 10.1017/S0140525X98001253 10097019

[b35] Epstein , R. A . ( 2008 ). Parahippocampal and retrosplenial contributions to human spatial navigation . Trends in Cognitive Sciences , 12 ( 10 ), 388 – 396 . 10.1016/j.tics.2008.07.004 18760955 PMC2858632

[b36] Esteban , O. , Markiewicz , C. J. , Blair , R. W. , Moodie , C. A. , Isik , A. I. , Erramuzpe , A. , Kent , J. D. , Goncalves , M. , DuPre , E. , Snyder , M. , Oya , H. , Ghosh , S. S. , Wright , J. , Durnez , J. , Poldrack , R. A. , & Gorgolewski , K. J . ( 2019 ). FMRIPrep: A robust preprocessing pipeline for functional MRI . FMRIPrep: A Robust Preprocessing Pipeline for Functional MRI , 16 ( 1 ), 1 – 29 . 10.1038/s41592-018-0235-4 PMC6319393 30532080

[b37] Fabbri , S. , Caramazza , A. , & Lingnau , A . ( 2010 ). Tuning curves for movement direction in the human visuomotor system . Journal of Neuroscience , 30 ( 40 ), 13488 – 13498 . 10.1523/JNEUROSCI.2571-10.2010 20926674 PMC6634734

[b38] Fernandino , L. , Tong , J. Q. , Conant , L. L. , Humphries , C. J. , & Binder , J. R . ( 2022 ). Decoding the information structure underlying the neural representation of concepts . Proceedings of the National Academy of Sciences of the United States of America , 119 ( 6 ), 1 – 11 . 10.1073/pnas.2108091119 PMC8832989 35115397

[b39] Formisano , E. , Kim , D. S. , Di Salle , F. , Van De Moortele , P. F. , Ugurbil , K. , & Goebel , R . ( 2003 ). Mirror-symmetric tonotopic maps in human primary auditory cortex . Neuron , 40 ( 4 ), 859 – 869 . 10.1016/S0896-6273(03)00669-X 14622588

[b40] Freud , E. , Culham , J. C. , Plaut , D. C. , & Behrmann , M . ( 2017 ). The large-scale organization of shape processing in the ventral and dorsal pathways . ELife , 6 , 1 – 26 . 10.7554/eLife.27576 PMC5659821 28980938

[b41] Freud , E. , Ganel , T. , Shelef , I. , Hammer , M. D. , Avidan , G. , & Behrmann , M . ( 2017 ). Three-dimensional representations of objects in dorsal cortex are dissociable from those in ventral cortex . Cerebral Cortex , 27 ( 1 ), 422 – 434 . 10.1093/cercor/bhv229 26483400 PMC13100970

[b42] Freud , E. , Plaut , D. C. , & Behrmann , M . ( 2016 ). ‘What’ is happening in the dorsal visual pathway . Trends in Cognitive Sciences , 20 ( 10 ), 773 – 784 . 10.1016/j.tics.2016.08.003 27615805

[b43] Fritsche , M. , Lawrence , S. J. D. , & de Lange , F. P . ( 2020 ). Temporal tuning of repetition suppression across the visual cortex . Journal of Neurophysiology , 123 ( 1 ), 224 – 233 . 10.1152/jn.00582.2019 31774368 PMC6985851

[b44] Gallivan , J. P. , Cant , J. S. , Goodale , M. A. , & Flanagan , J. R . ( 2014 ). Representation of object weight in human ventral visual cortex . Current Biology , 24 ( 16 ), 1866 – 1873 . 10.1016/j.cub.2014.06.046 25065755

[b45] Garcea , F. E. , Kristensen , S. , Almeida , J. , & Mahon , B. Z . ( 2016 ). Resilience to the contralateral visual field bias as a window into object representations . Cortex , 81 , 14 – 23 . 10.1016/j.cortex.2016.04.006 27160998 PMC4958537

[b46] Georgieva , S. S. , Todd , J. T. , Peeters , R. , & Orban , G. A . ( 2008 ). The extraction of 3D shape from texture and shading in the human brain . Cerebral Cortex , 18 ( 10 ), 2416 – 2438 . 10.1093/cercor/bhn002 18281304 PMC2536698

[b47] Gotts , S. J. , Milleville , S. C. , Bellgowan , P. S. F. , & Martin , A . ( 2011 ). Broad and narrow conceptual tuning in the human frontal lobes . Cerebral Cortex , 21 ( 2 ), 477 – 491 . 10.1093/cercor/bhq113 20562319 PMC3020586

[b48] Greve , D. N. , & Fischl , B . ( 2009 ). Accurate and robust brain image alignment using boundary-based registration . NeuroImage , 48 ( 1 ), 63 – 72 . 10.1016/j.neuroimage.2009.06.060 19573611 PMC2733527

[b49] Grill-Spector , K. , Henson , R. , & Martin , A . ( 2006 ). Repetition and the brain: Neural models of stimulus-specific effects . Trends in Cognitive Sciences , 10 ( 1 ), 14 – 23 . 10.1016/j.tics.2005.11.006 16321563

[b50] Grill-Spector , K. , Kushnir , T. , Edelman , S. , Avidan , G. , Itzchak , Y. , & Malach , R . ( 1999 ). Differential processing of objects under various viewing conditions in the human lateral occipital complex . Neuron , 24 ( 1 ), 187 – 203 . 10.1016/S0896-6273(00)80832-6 10677037

[b51] Grill-Spector , K. , & Malach , R . ( 2001 ). fMR-adaptation: A tool for studying the functional properties of human cortical neurons . Acta Psychologica , 107 ( 1–3 ), 293 – 321 . 10.1016/S0001-6918(01)00019-1 11388140

[b52] Grill-Spector , K. , & Weiner , K. S . ( 2014 ). The functional architecture of the ventral temporal cortex and its role in categorization . Nature Reviews Neuroscience , 15 ( 8 ), 536548 . 10.1038/nrn3747 PMC4143420 24962370

[b53] Harvey , B. M. , Klein , B. P. , Petridou , N. , & Dumoulin , S. O . ( 2013 ). Topographic representation of numerosity in the human parietal cortex . Science , 341 ( 6150 ), 1123 – 1126 . 10.1126/science.1239052 24009396

[b54] Haxby , J. V , Gobbini , M. I. , Furey , M. L. , Ishai , A. , Schouten , J. L. , & Pietrini , P . ( 2001 ). Distributed and overlapping representations of faces and objects in ventral temporal cortex . Science , 293 ( 5539 ), 2425 – 2430 . 10.1126/science.1063736 11577229

[b55] Hayworth , K. J. , & Biederman , I . ( 2006 ). Neural evidence for intermediate representations in object recognition . Vision Research , 46 ( 23 ), 4024 – 4031 . 10.1016/j.visres.2006.07.015 16979693

[b56] Hebart , M. N. , Zheng , C. Y. , Pereira , F. , & Baker , C. I . ( 2020 ). Revealing the multidimensional mental representations of natural objects underlying human similarity judgments . Nature Human Behaviour , 4 ( 11 ), 1173 – 1185 . 10.1038/s41562-020-00951-3 PMC7666026 33046861

[b57] Huth , A. G. , Nishimoto , S. , Vu , A. T. , & Gallant , J. L . ( 2012 ). A continuous semantic space describes the representation of thousands of object and action categories across the human brain . Neuron , 76 ( 6 ), 1210 – 1224 . 10.1016/j.neuron.2012.10.014 23259955 PMC3556488

[b58] Jeannerod , M. , Decety , J. , & Michel , F . ( 1994 ). Impairment of grasping movements following a bilateral posterior parietal lesion . Neuropsychologia , 32 ( 4 ), 369 – 380 . 10.1016/0028-3932(94)90084-1 8047246

[b59] Jenkinson , M. , Beckmann , C. F. , Behrens , T. E. J. , Woolrich , M. W. , & Smith , S. M . ( 2012 ). FSL . NeuroImage , 62 ( 2 ), 782 – 790 . 10.1016/J.NEUROIMAGE.2011.09.015 21979382

[b60] Kanwisher , N. , Mcdermott , J. , & Chun , M. M . ( 1997 ). The fusiform face area: A module in human extrastriate cortex specialized for face perception . The Journal of Neuroscience , 17 ( 11 ), 4302 – 4311 . 10.1523/jneurosci.17-11-04302.1997 9151747 PMC6573547

[b61] Kasper , L. , Bollmann , S. , Diaconescu , A. O. , Hutton , C. , Heinzle , J. , Iglesias , S. , Hauser , T. U. , Sebold , M. , Manjaly , Z.-M. , Pruessmann , K. P. , & Stephan , K. E . ( 2017 ). The PhysIO Toolbox for modeling physiological noise in fMRI data . Journal of Neuroscience Methods , 276 , 56 – 72 . 10.1016/j.jneumeth.2016.10.019 27832957

[b62] Kersey , A. J. , & Cantlon , J. F . ( 2017 ). Neural tuning to numerosity relates to perceptual tuning in 3–6-year-old children . Journal of Neuroscience , 37 ( 3 ), 512 – 522 . 10.1523/JNEUROSCI.0065-16.2016 28100735 PMC5242404

[b63] Kilintari , M. , Raos , V. , & Savaki , H. E . ( 2011 ). Grasping in the dark activates early visual cortices . Cerebral Cortex , 21 ( 4 ), 949 – 963 . 10.1093/cercor/bhq175 20833697

[b64] King , M. L. , Groen , I. I. A. , Steel , A. , Kravitz , D. J. , & Baker , C. I . ( 2019 ). Similarity judgments and cortical visual responses reflect different properties of object and scene categories in naturalistic images . NeuroImage , 197 ( October 2018 ), 368 – 382 . 10.1016/j.neuroimage.2019.04.079 31054350 PMC6591094

[b65] Klein , A. , Ghosh , S. S. , Bao , F. S. , Giard , J. , Häme , Y. , Stavsky , E. , Lee , N. , Rossa , B. , Reuter , M. , Neto , E. C. , Keshavan , A. , & Keshavan , A . ( 2017 ). Mindboggling morphometry of human brains . PLoS Computational Biology , 13 ( 2 ), e1005350 . 10.1371/journal.pcbi.1005350 28231282 PMC5322885

[b66] Kleineberg , N. N. , Dovern , A. , Binder , E. , Grefkes , C. , Eickhoff , S. B. , Fink , G. R. , & Weiss , P. H . ( 2018 ). Action and semantic tool knowledge—Effective connectivity in the underlying neural networks . Human Brain Mapping , 39 ( 9 ), 3473 – 3486 . 10.1002/hbm.24188 29700893 PMC6866288

[b67] Knights , E. , Smith , F. W. , & Rossit , S . ( 2022 ). The role of the anterior temporal cortex in action: Evidence from fMRI multivariate searchlight analysis during real object grasping . Scientific Reports , 12 ( 9042 ), 1 – 10 . 10.1038/s41598-022-12174-9 35662252 PMC9167815

[b68] Konkle , T. , & Caramazza , A . ( 2013 ). Tripartite organization of the ventral stream by animacy and object size . Journal of Neuroscience , 33 ( 25 ), 10235 – 10242 . 10.1523/JNEUROSCI.0983-13.2013 23785139 PMC3755177

[b69] Kriegeskorte , N. , Mur , M. , Ruff , D. A. , Kiani , R. , Bodurka , J. , Esteky , H. , & Bandettini , P. A . ( 2008 ). Matching categorical object representations in inferior temporal cortex of man and monkey . Neuron , 60 ( 6 ), 1126 – 1141 . 10.1016/j.neuron.2008.10.043 19109916 PMC3143574

[b70] Kriegeskorte , N. , Simmons , W. K. , Bellgowan , P. S. F. , & Baker , C. I . ( 2009 ). Circular analysis in systems neuroscience: The dangers of double dipping . Nature Neuroscience , 12 ( 5 ), 535 – 540 . 10.1038/nn.2303 19396166 PMC2841687

[b71] Kristensen , S. , Garcea , F. E. , Mahon , B. Z. , & Almeida , J . ( 2016 ). Temporal frequency tuning reveals interactions between the dorsal and ventral visual streams . Journal of Cognitive Neuroscience , 28 ( 9 ), 1295 – 1302 . 10.1162/jocn_a_00969 27082048 PMC5193157

[b72] Larsson , J. , Solomon , S. G. , & Kohn , A . ( 2016 ). fMRI adaptation revisited . Cortex , 80 , 154 – 160 . 10.1016/j.cortex.2015.10.026 26703375 PMC4870150

[b73] Lee , D. , Mahon , B. , & Almeida , J . ( 2019 ). Action at a distance on object-related ventral temporal representations . Cortex , 117 , 157 – 167 . 10.1016/j.cortex.2019.02.018 30981039

[b74] Levy , I. , Hasson , U. , Avidan , G. , Hendler , T. , & Malach , R . ( 2001 ). Center-periphery organization of human object areas . Nature Neuroscience , 4 ( 5 ), 533 – 539 . 10.1038/87490 11319563

[b75] Lingnau , A. , & Downing , P. E . ( 2015 ). The lateral occipitotemporal cortex in action . Trends in Cognitive Sciences , 19 ( 5 ), 268 – 277 . 10.1016/j.tics.2015.03.006 25843544

[b76] Mahon , B. Z. , & Almeida , J . ( 2024 ). Reciprocal interactions between parietal and occipito-temporal representations support everyday object-directed actions . Neuropsychologia , 198 ( 108841 ), 1 – 13 . 10.1016/j.neuropsychologia.2024.108841 PMC11498102 38430962

[b77] Mahon , B. Z. , & Caramazza , A . ( 2011 ). What drives the organization of object knowledge in the brain? Trends in Cognitive Sciences , 15 ( 3 ), 97 – 103 . 10.1016/j.tics.2011.01.004 21317022 PMC3056283

[b78] Mahon , B. Z. , Kumar , N. , & Almeida , J . ( 2013 ). Spatial frequency tuning reveals interactions between the dorsal and ventral visual systems . Journal of Cognitive Neuroscience , 25 ( 6 ), 862 – 871 . 10.1162/jocn_a_00370 23410033 PMC3767423

[b79] Mahon , B. Z. , Milleville , S. C. , Negri , G. A. L. , Rumiati , R. I. , Caramazza , A. , & Martin , A . ( 2007 ). Action-related properties shape object representations in the ventral stream . Neuron , 55 ( 3 ), 507 – 520 . 10.1016/j.neuron.2007.07.011 17678861 PMC2000824

[b80] Mandera , P. , Keuleers , E. , & Brysbaert , M . ( 2017 ). Explaining human performance in psycholinguistic tasks with models of semantic similarity based on prediction and counting: A review and empirical validation . Journal of Memory and Language , 92 , 57 – 78 . 10.1016/j.jml.2016.04.001

[b81] Monaco , S. , Cavina-Pratesi , C. , Sedda , A. , Fattori , P. , Galletti , C. , & Culhaml , J. C . ( 2011 ). Functional magnetic resonance adaptation reveals the involvement of the dorsomedial stream in hand orientation for grasping . Journal of Neurophysiology , 106 ( 5 ), 2248 – 2263 . 10.1152/jn.01069.2010 21795615

[b82] Moss , H. E. , & Tyler , L. K . ( 2000 ). A progressive category-specific semantic deficit for non-living things . Neuropsychologia , 38 , 60 – 82 . 10.1016/s0028-3932(99)00044-5 10617292

[b83] Op De Beeck , H. P. , Haushofer , J. , & Kanwisher , N. G . ( 2008 ). Interpreting fMRI data: Maps, modules and dimensions . Nature Reviews Neuroscience , 9 ( 2 ), 123 – 135 . 10.1038/nrn2314 18200027 PMC2731480

[b84] Peuskens , H. , Claeys , K. G. , Todd , J. T. , Norman , J. F. , Van Hecke , P. , & Orban , G. A . ( 2004 ). Attention to 3-D shape, 3-D motion, and texture in 3-D structure from motion displays . Journal of Cognitive Neuroscience , 16 ( 4 ), 665 – 682 . 10.1162/089892904323057371 15165355

[b85] Piazza , M. , Izard , V. , Pinel , P. , Le Bihan , D. , & Dehaene , S . ( 2004 ). Tuning curves for approximate numerosity in the human intraparietal sulcus . Neuron , 44 ( 3 ), 547 – 555 . 10.1016/j.neuron.2004.10.014 15504333

[b86] Piazza , M. , Pinel , P. , Le Bihan , D. , & Dehaene , S . ( 2007 ). A magnitude code common to numerosities and number symbols in human intraparietal cortex . Neuron , 53 ( 2 ), 293 – 305 . 10.1016/j.neuron.2006.11.022 17224409

[b87] Planton , S. , van Kerkoerle , T. , Abbih , L. , Maheu , M. , Meyniel , F. , Sigman , M. , & Dehaene , S . ( 2021 ). A theory of memory for binary sequences: Evidence for a mental compression algorithm in humans . PLoS Computational Biology , 17 ( 1 ), e1008598 . 10.1371/journal.pcbi.1008598 33465081 PMC7845997

[b88] Pruim , R. H. R. , Mennes , M. , van Rooij , D. , Llera , A. , Buitelaar , J. K. , & Beckmann , C. F . ( 2015 ). ICA-AROMA: A robust ICA-based strategy for removing motion artifacts from fMRI data . NeuroImage , 112 , 267 – 277 . 10.1016/j.neuroimage.2015.02.064 25770991

[b89] Pyles , J. A. , & Grossman , E. D . ( 2009 ). Neural adaptation for novel objects during dynamic articulation . Neuropsychologia , 47 ( 5 ), 1261 – 1268 . 10.1016/j.neuropsychologia.2009.01.006 19428389

[b90] Reuter , M. , Rosas , H. D. , & Fischl , B . ( 2010 ). Highly accurate inverse consistent registration: A robust approach . NeuroImage , 53 ( 4 ), 1181 – 1196 . 10.1016/j.neuroimage.2010.07.020 20637289 PMC2946852

[b91] Rosch , E. , Mervis , C. B. , Gray , W. D. , Johnson , D. M. , & Boyes-Braem , P . ( 1976 ). Basic objects in natural categories . Cognitive Psychology , 8 ( 3 ), 382 – 439 . 10.1016/0010-0285(76)90013-X

[b92] Ruotolo , F. , Kalénine , S. , & Bartolo , A . ( 2020 ). Activation of manipulation and function knowledge during visual search for objects . Journal of Experimental Psychology: Human Perception and Performance , 46 ( 1 ), 66 – 90 . 10.1037/xhp0000696 31580140

[b93] Satterthwaite , T. D. , Elliott , M. A. , Gerraty , R. T. , Ruparel , K. , Loughead , J. , Calkins , M. E. , & Wolf , D. H . ( 2013 ). An improved framework for confound regression and filtering for control of motion artifact in the preprocessing of resting-state functional connectivity data . NeuroImage , 64 ( 1 ), 1 – 39 . 10.1016/j.neuroimage.2012.08.052 22926292 PMC3811142

[b94] Schwarzbach , J . ( 2011 ). A simple framework (ASF) for behavioral and neuroimaging experiments based on the psychophysics toolbox for MATLAB . Behavior Research Methods , 43 ( 4 ), 1194 – 1201 . 10.3758/s13428-011-0106-8 21614662

[b95] Sereno , M. I. , Dale , A. M. , Reppas , J. B. , Kwong , K. K. , Belliveau , J. W. , Brady , T. J. , & Tootell , R. B. H . ( 1995 ). Borders of multiple visual areas in humans revealed by functional MRI . Science , 268 , 889 – 893 . 10.1126/science.7754376 7754376

[b96] Sha , L. , Haxby , J. V , Abdi , H. , Swaroop Guntupalli , J., Oosterhof , N. N. , Halchenko , Y. O. , & Connolly , A. C . ( 2015 ). The animacy continuum in the human ventral vision pathway . Journal of Cognitive Neuroscience , 27 ( 4 ), 665 – 678 . 10.1162/jocn_a_00733 25269114

[b97] Stevens , D. W. , Tessler , M. H. , Peng , C. S. , & Martin , A . ( 2015 ). Functional connectivity constrains the category-related organization of human ventral occipitotemporal cortex . Human Brain Mapping , 36 ( 6 ), 2187 – 2206 . 10.1002/hbm.22764 25704493 PMC4414790

[b98] Sun , H. C. , Ban , H. , Di Luca , M. , & Welchman , A. E . ( 2015 ). FMRI evidence for areas that process surface gloss in the human visual cortex . Vision Research , 109 , 149 – 157 . 10.1016/j.visres.2014.11.012 25490434 PMC4410797

[b99] Tustison , N. J. , Avants , B. B. , Cook , P. A. , Zheng , Y. , Egan , A. , Yushkevich , P. A. , & Gee , J. C . ( 2010 ). N4ITK: Improved N3 bias correction . IEEE Transactions on Medical Imaging , 29 ( 6 ), 1310 – 1320 . 10.1109/TMI.2010.2046908 20378467 PMC3071855

[b100] Valério , D. , Hussain , A. , & Almeida , J . ( 2023 ). Semantic feature production norms for manipulable objects . Cognitive Neuropsychology , 40 ( 3–4 ), 167 – 185 . 10.1080/02643294.2023.2279185 38006205

[b101] Valyear , K. F. , & Culham , J. C . ( 2010 ). Observing learned object-specific functional grasps preferentially activates the ventral stream . Journal of Cognitive Neuroscience , 22 ( 5 ), 970 – 984 . 10.1162/jocn.2009.21256 19413481

[b102] Walbrin , J. , & Almeida , J . ( 2021 ). High-level representations in human occipito-temporal cortex are indexed by distal connectivity . The Journal of Neuroscience , 41 ( 21 ), 4678 – 4685 . 10.1523/JNEUROSCI.2857-20.2021 33849949 PMC8260247

[b103] Wheatley , T. , Weisberg , J. , Beauchamp , M. S. , & Martin , A . ( 2005 ). Automatic priming of semantically related words reduces activity in the fusiform gyrus . Journal of Cognitive Neuroscience , 17 ( 12 ), 1871 – 1885 . 10.1162/089892905775008689 16356325

[b104] Wurm , M. F. , & Lingnau , A . ( 2015 ). Decoding actions at different levels of abstraction . Journal of Neuroscience , 35 ( 20 ), 7727 – 7735 . 10.1523/JNEUROSCI.0188-15.2015 25995462 PMC6795187

[b105] Xu , Y. , Vignali , L. , Sigismondi , F. , Crepaldi , D. , Bottini , R. , & Collignon , O . ( 2023 ). Similar object shape representation encoded in the inferolateral occipitotemporal cortex of sighted and early blind people . PLoS Biology , 21 ( 7 ), 1 – 36 . 10.1371/journal.pbio.3001930 PMC10368275 37490508

[b106] Zhang , Y. , Brady , M. , & Smith , S . ( 2001 ). Segmentation of brain MR images through a hidden Markov random field model and the expectation-maximization algorithm . IEEE Transactions on Medical Imaging , 20 ( 1 ), 45 – 57 . 10.1109/42.906424 11293691

