# Supplementary material for: Neural and behavioral similarity-driven tuning curves for manipulable objects
Source: Imaging Neurosci (Camb). 2025 Feb 18;3:imag_a_00482. doi: 10.1162/imag_a_00482 (PMC12319753; doi:10.1162/imag_a_00482)
Supplement: Supplementary Material [file imag_a_00482-supp.pdf]

Supplementary Material for

Neural and behavioral tuning curves for object similarity

Valério, D.[1,2], Peres, A. [1,2], Bergström, F.[1,2,3], Seidel, P.[4], & Almeida, J.[1,2*]

Supplementary Table S1: Pairs used in the Behavioral Experiments, in brackets there is the cosine similarity between adaptor and each deviant.

Supplementary Figure S1: Reaction Times (in ms) per deviant type and participant in A) Experiment 1a and B) Experiment 1b.

Supplementary Figure S2: Areas presenting a difference between the deviants and the Identity condition and their relationship with the areas that present release from adaptation.

Supplementary Figure S3: fMRI adaptation.

Supplementary Figure S4: Areas that exhibit adaptation and release from adaptation as a function of similarity between adaptation and deviant objects without excluding the outlier.

Supplementary Figure S5: Areas that exhibited release across the whole brain (*p*FWE-corrected < 0.001).

Supplementary Table S1: Pairs used in the Behavioral Experiments, in brackets there is the cosine similarity between adaptor and each deviant.

	Adaptors	Deviants			
		SC	C	D	SD
Experiment 1a	Hoe	shovel (0.76)	plunger (0.41)	door handle (0.10)	cork (0.01)
	Drill	drill bit (0.74)	hole punch (0.46)	paint brush (0.10)	hanger (0.03)
	Scissors	knife (0.75)	grater (0.47)	sponge (0.09)	match (0.06)
	Fork	spoon (0.71)	peeler (0.41)	match (0.10)	cork (0.06)
	Pliers	swiss knife (0.67)	screw (0.42)	board eraser (0.10)	napkin (0.06)
	Bucket	bottle (0.64)	bowl (0.42)	nutcracker (0.09)	horn (0.03)
	Glass	bottle (0.90)	cup (0.54)	weights (0.10)	horn (0.03)
	Bottle Cap	cork (0.76)	bottle opener (0.43)	weights (0.10)	candle holder (0.04)
	Hand-Blender	grater (0.78)	peeler (0.42)	mop (0.10)	candle holder (0.06)
	Stapler	paper clip (0.75)	rubber stamp (0.41)	spinning top (0.10)	plunger (0.06)
Experiment 1b	Shovel	hoe (0.76)	mop (0.6)	bottle opener (0.27)	sponge (0.08)
	Knife	scissors (0.75)	wooden spoon (0.40)	pastry bag (0.21)	pencil eraser (0.08)
	Bottle	glass (0.90)	bowl (0.51)	citrus squeezer (0.25)	horn (0.02)
	Broom	mop (0.76)	plunger (0.54)	pencil (0.20)	door handle (0.06)
	Spoon	fork (0.71)	peeler (0.42)	clothespin (0.19)	shaver (0.07)
	Grater	hand blender (0.78)	pliers (0.40)	hammer (0.21)	board eraser (0.07)
	Cork	bottle cap (0.76)	bottle opener (0.31)	rolling pin (0.10)	candle holder (0.01)
	Swiss Knife	knife (0.71)	grater (0.42)	plunger (0.20)	napkin (0.06)
	Nail clipper	scissors (0.64)	shaver (0.38)	hammer (0.17)	paddle (0.04)
	Paper clip	stapler (0.75)	screw (0.41)	clothespin (0.18)	paddle (0.03)

Supplementary Table S2: The mean (M) of features per object and the standard deviation (SD) for the different categories of features in the deviant conditions of both Experiments 1a and 1b.

	Super Close	Close	Distant	Super Distant
Visual	M = 10.9; SD=3.1	M= 10.4; SD=2.7	M=9.4; SD=2.08	M=8; SD=2.1
Encyclopedic	M=6.2; SD=2.4	M=7; SD=1.4	M=6.7; SD=2.5	M=7.3; SD=2.1
Function	M=5.8; SD=2.5	M=5.3; SD=1.5	M=4.8; SD=1.2	M=4.8; SD=1.5
Taxonomic	M=2.5; SD=1.1	M=2.3; SD=1.3	M=1.7; SD=0.73	M=1; SD=0.4
Action	M=1.6; SD=0.9	M=1.5; SD=0.6	M=2.4; SD=0.92	M=1.7; SD=1.2
Tactile	M=1.4; SD=0.7	M=1.2; SD=0.4	M=1.7; SD=0.9	M=1; SD=0.6
Sound	M=0.1; SD=0.3	M=0.1; SD=0.2	M=0.1; SD=0.3	M=0.2; SD=0.4

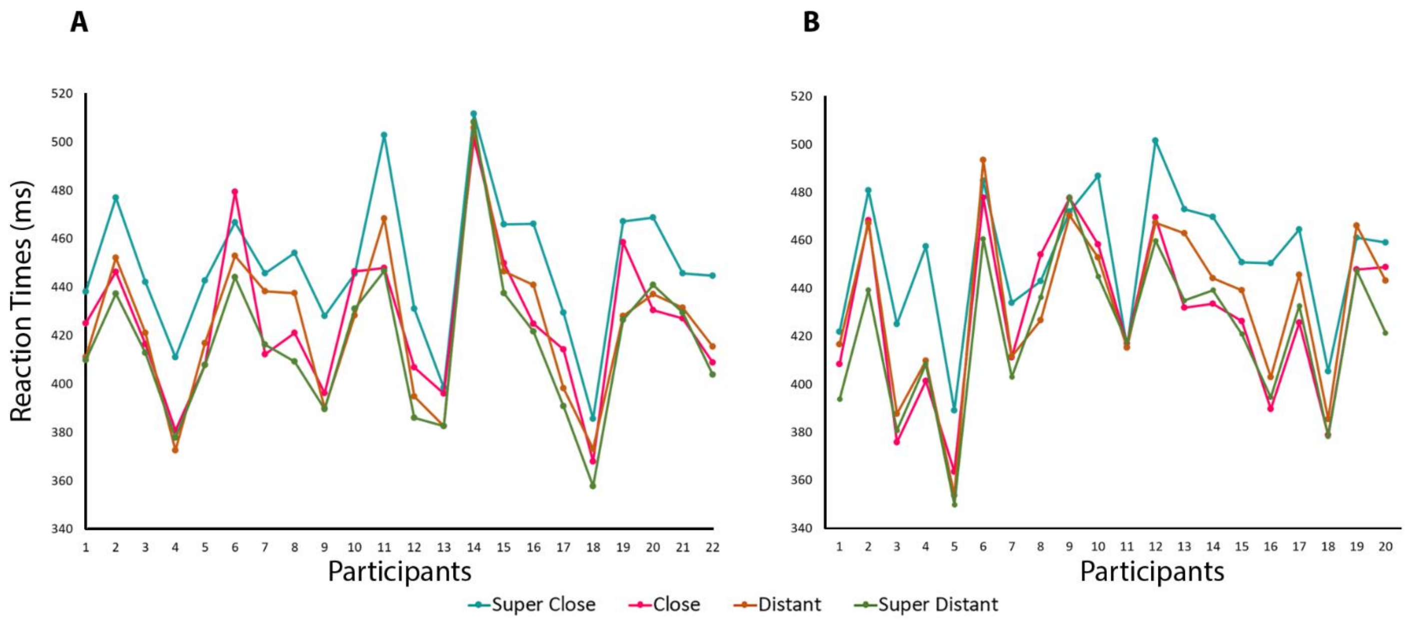

Supplementary Figure S1: Reaction Times (in ms) per deviant type and participant in A) Experiment 1a and B) Experiment 1b. The blue line represents the Super Close condition, the pink line represents the Close condition, orange represents Distant, and green represents Super Distant conditions.

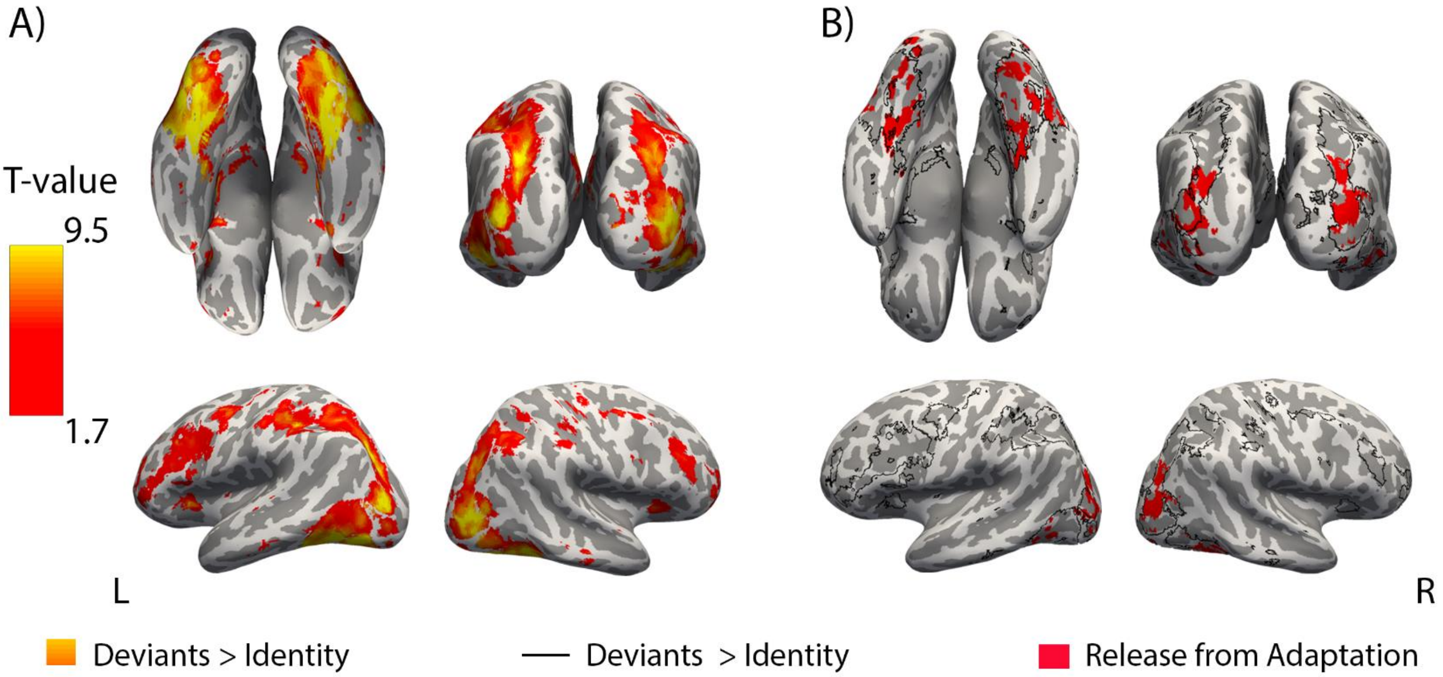

Supplementary Figure S2: Areas presenting a difference between the deviants and the Identity condition and their relationship with the areas that present release from adaptation. **A)** A paired t-test was conducted between the four deviants (i.e., SC, C, D, and SD) and the Identity condition, $p < 0.05$ (uncorrected). **B)** The figure illustrates the overlap between the contrast Deviants > Identity (in black outline) and the areas that have a release from adaptation (in red).

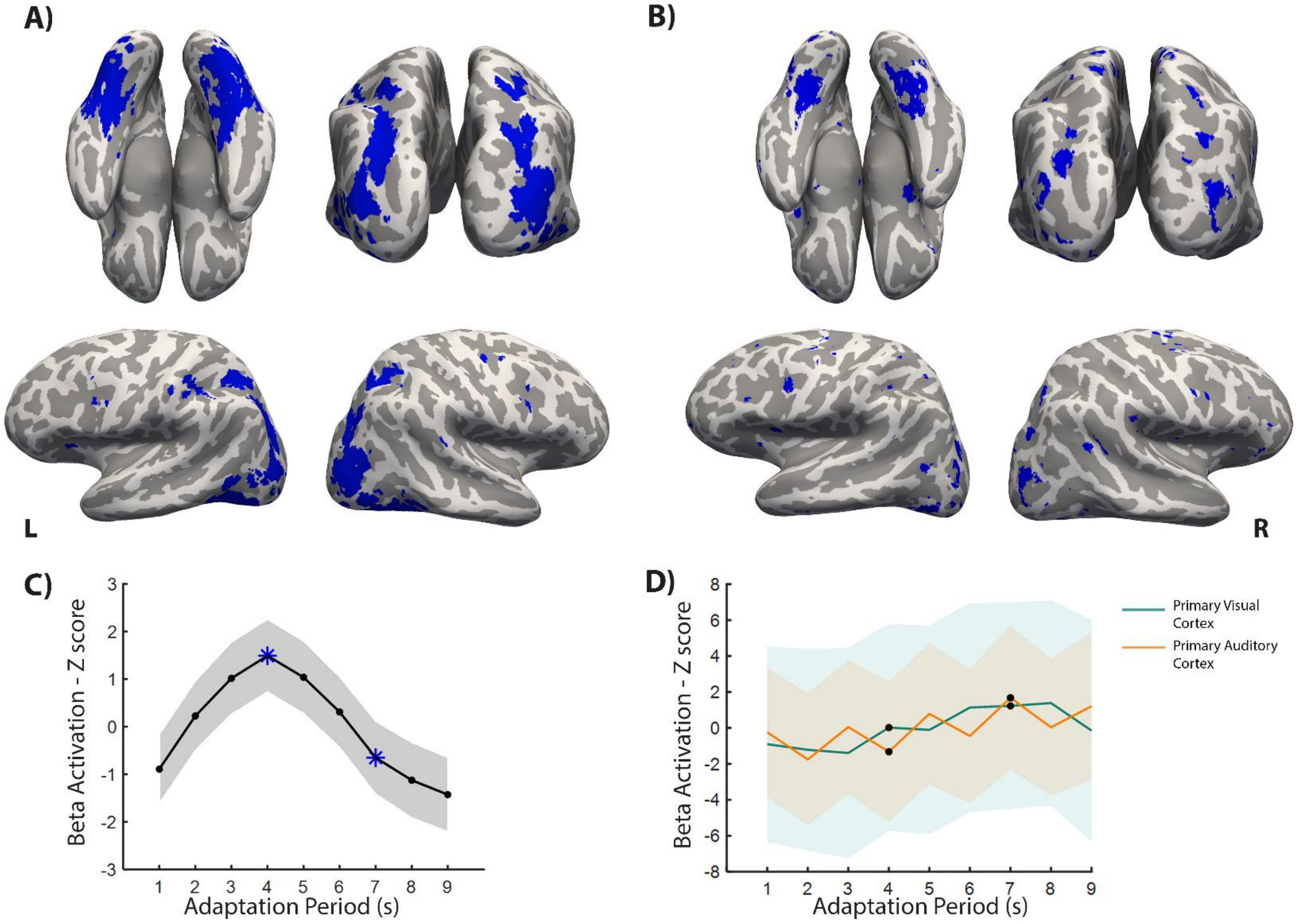

Supplementary Figure S3: fMRI-adaptation. A) Areas that presented adaptation defined as $4^{th} > 7^{th}$ FIR time bins under $p < 0.001$ (uncorrected). These areas are presented in the paper in Figure 3A, blue outline. B) Areas showing adaptation, defined as $1^{st} > 9^{th}$ FIR time bins, under $p < 0.05$ (uncorrected). C) The line graph represents the BOLD adaptation effect (Z-score and SEM) as a function of the nine FIR time bins. The blue asterisks show the two moments that we used to define adaptation in the main analysis. D) The line graph shows the BOLD response (Z-score and SEM) as a function of the nine FIR time bins for the Primary Visual Cortex and the Primary Auditory Cortex, areas that did not exhibit adaptation. The ROIs were extracted from NeuroSynth (term-based meta-analyses), retaining the 200 most activated voxels for each hemisphere.

We evaluated the characteristics of the two neuronal tuning curves in the regions that showed release from adaptation (see Figure S4). Without excluding the outlier participant, we compared the slope of cluster 1 (M = 0.16; SEM = 0.11) and cluster 2 (M = 0.08; SEM = 0.07) using a paired t-test, but it did not reach statistical significance ($t(20) = 1.07$, $p = 0.15$, one-tailed).

We conducted paired t-tests (two-tailed) to compare beta values across deviants for each cluster. In cluster 1, beta activation values are statistically lower for SC (M = -0.08, SEM = 0.74) than C (M = 1.01, SEM = 0.80; $t(20) = -3.01$, $p = 0.007$). Other comparisons were not statistically significant after Bonferroni correction, such as SC and SD (M = 0.70; SEM = 0.64; $t(20) =-2.37$, $p = 0.03$, see Figure S4C); SC and D (M = 0.23; SEM = 0.74; $t(20) = -0.89$, $p = 0.39$), C and D ($t(20) = 2.36$, $p = 0.03$), C and SD ($t(20) = 0.70$, $p = 0.49$), and D and SD ($t(20) = -1.18$, $p = 0.25$). Using a similar approach, in cluster 2, we did not find any statistically significant results after Bonferroni correction: SC (M = -0.07; SEM = 0.65) and C (M = 0.94; SEM = 0.63; $t(20) = -2.66$, $p = 0.02$; see Figure S4C), SC and D (M = 0.73; SEM = 0.62; $t(20) = -2.41$, $p = 0.03$), SC and SD (M = 0.27, SEM = 0.60; $t(20) = -1.30$, $p = 0.21$), C and D ($t(20) = 0.79$, $p = 0.44$), C and SD ($t(20) = 2.25$, $p = 0.04$), and D and SD ($t(20) = 1.43$, $p = 0.17$).

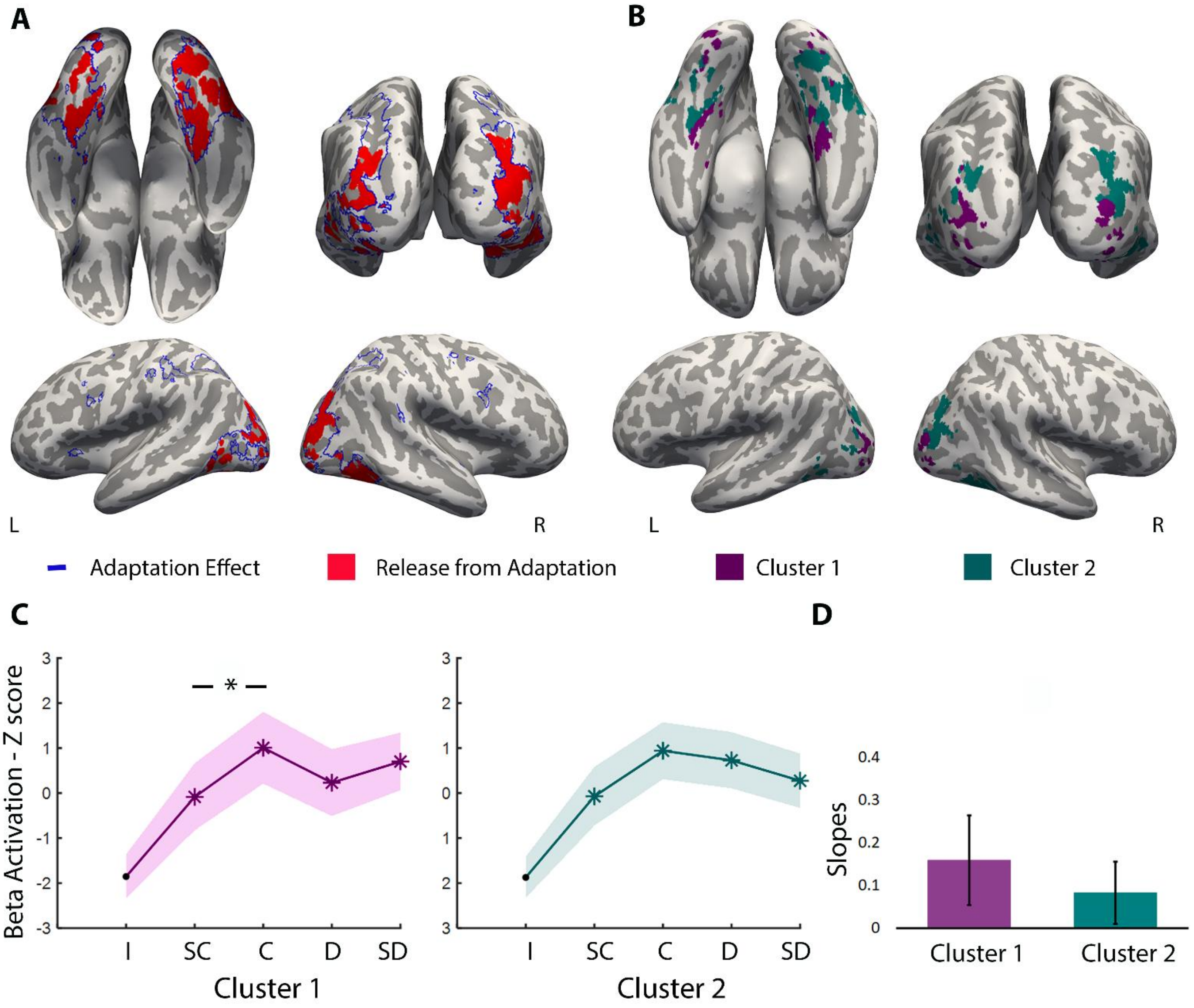

Supplementary Figure S4: Areas that exhibit adaptation and release from adaptation as a function of similarity between adaptation and deviant objects without excluding the outlier. A) The blue outline encompasses areas that exhibit adaptation ($4^{th} > 7^{th}$ FIR time bins) at a threshold of $p < 0.001$ (uncorrected). Those areas were within ventral occipitotemporal cortex (that goes from LOC to parahippocampal gyrus), parietal lobe (inferior and superior) and occipito-parietal cortex, and frontal lobe (superior, middle, and inferior frontal gyrus, and supplementary motor area). In red, we present areas that show release from adaptation as a function of object similarity (pFWE-corrected < 0.001). Bilaterally, these include the collateral sulcus and fusiform gyrus (posterior to anterior), the parahippocampal gyrus, the lingual gyrus, the inferior temporal gyrus, the middle temporal gyrus, LOC and the most posterior part of the parietal lobe and occipito-parietal cortex. **B)** Here, we show the areas corresponding to the two clusters that are present in at least 16 participants (75% of our sample). Cluster 1 comprises LOC, occipito-parietal cortex, lingual gyrus, parahippocampal, collateral sulcus and the most anterior region of medial fusiform; Cluster 2 comprises parts of the fusiform gyrus, middle temporal gyrus, inferior temporal gyrus, and bilateral occipito-parietal cortex and posterior/caudal IPS. **C)** The graphs represent

the release effect in BOLD activation (Z-score and SEM) as a function of the four deviants for the areas of cluster 1 (in purple) and cluster 2 (in green). Identity (I) condition was never used in our analysis, presented here to show that beta activation is below the main conditions in the two clusters. **D)** The graph illustrates the differences in slopes between clusters 1 and 2.

Supplementary Table S3: Paired t-test between adaptation objects and their correspondent deviants (SC, C, D, and SD), for Experiment 1a and 2.

	HSF	Luminance	Contrast
Adaptation – SC	t(9) = -0.38, p = 0.71	t(9) = -0.41, p = 0.70	t(9) = 0.47, p = 0.65
Adaptation – C	t(9) = 2.46, p = 0.04	t(9) = 1.53, p = 0.16	t(9) = -1.44, p = 0.18
Adaptation – D	t(9) = 0.34, p = 0.68	t(9) = -0.19, p = 0.85	t(9) = 0.49, p = 0.64
Adaptation - SD	t(9) = 0.43, p = 0.86	t(9) = -0.81, p = 0.44	t(9) = 0.38, p = 0.72

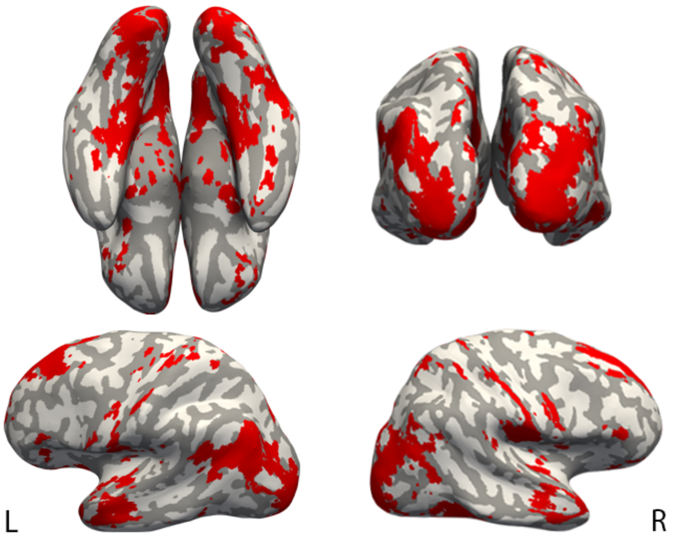

Supplementary Figure S5: Areas that exhibited release across the whole brain (*p*FWE-corrected < 0.001). Bilaterally, these include the collateral sulcus and fusiform gyrus (posterior to anterior), the parahippocampal gyrus, hippocampi, the lingual gyrus, the inferior temporal gyrus, the middle temporal gyrus, LOC, parietal lobe, premotor cortex, and the inferior, middle and superior frontal gyri, as well as paracingulate and cingulate gyri.
